# Gut microbiota and metabolomics reveal the combined effects of *Alpinia oxyphylla* fructus polysaccharide and nootkatone on chronic kidney disease via the “gut-kidney” axis

**DOI:** 10.3389/fphar.2026.1904155

**Published:** 2026-09-08

**Authors:** Rong Zhang, Yangmei Chen, Haisen Meng, An Jia, Dingwen Fu, Xingzhou Lin, Jingzhe Wang, Jiajia He, Yue Zhou, Yonghui Li

**Affiliations:** 1 Engineering Research Center of Tropical Medicine Innovation and Transformation of Ministry of Education, Hainan Provincial Key Laboratory of Research and Development on Tropical Herbs, School of Pharmacy, Hainan Medical University, Haikou, China; 2 The Fifth People’s Hospital of Hainan Province, Haikou, China; 3 Medical School, Huanghe Science & Technology College, Zhengzhou, China

**Keywords:** *Alpinia oxyphylla* fructus polysaccharide, chronic kidney disease, gut microbiome, metabolomics, nootkatone

## Abstract

**Background:**

Chronic kidney disease (CKD) imposes a substantial burden on healthcare systems worldwide. Traditional Chinese medicine has gained attention as a complementary and alternative selection given the limitations of current CKD management. Although the renoprotective potential of *Alpinia oxyphylla* fructus has been reported, existing studies mainly focus on crude extracts or individual metabolites, leaving interactions among metabolites unexplored. This study investigated the individual and combined effects of *A. oxyphylla* fructus polysaccharide (AOP) and nootkatone (NKT) in CKD.

**Methods:**

The protective effects of AOP and NKT on CKD were evaluated using an adenine-induced mouse model. Renal and colonic injuries were assessed by plasma biochemistry, histopathological analysis, and inflammation-related gene expression. Plasma metabolic profiles were characterized by untargeted metabolomics. Intestinal microbiota structure was analyzed by microbial diversity sequencing. Fecal short-chain fatty acids (SCFAs) were quantified by gas chromatography, and intestinal barrier-related gene and protein expression levels were examined by RT-qPCR and immunohistochemistry, respectively.

**Results:**

Combined intervention exerted stronger protective effects than either intervention administered alone. It markedly reduced plasma urea nitrogen and creatinine levels, alleviated renal inflammation and fibrosis, and improved metabolic dysregulation with reduced uremic toxin accumulation. Key regulated pathways included pyrimidine, purine, and ascorbate and aldarate metabolism. The combined intervention also ameliorated gut microbiota dysbiosis, preserved SCFA levels, and maintained intestinal barrier integrity.

**Conclusion:**

The combination of AOP and NKT exhibited combined effects through modulation of the plasma metabolic profile and the gut microenvironment, suggesting its potential protective role against CKD via the gut-kidney axis. These findings provide new insights into the combined effects of multiple bioactive metabolites from traditional Chinese medicine.

## Graphical Abstract

**Figure:**
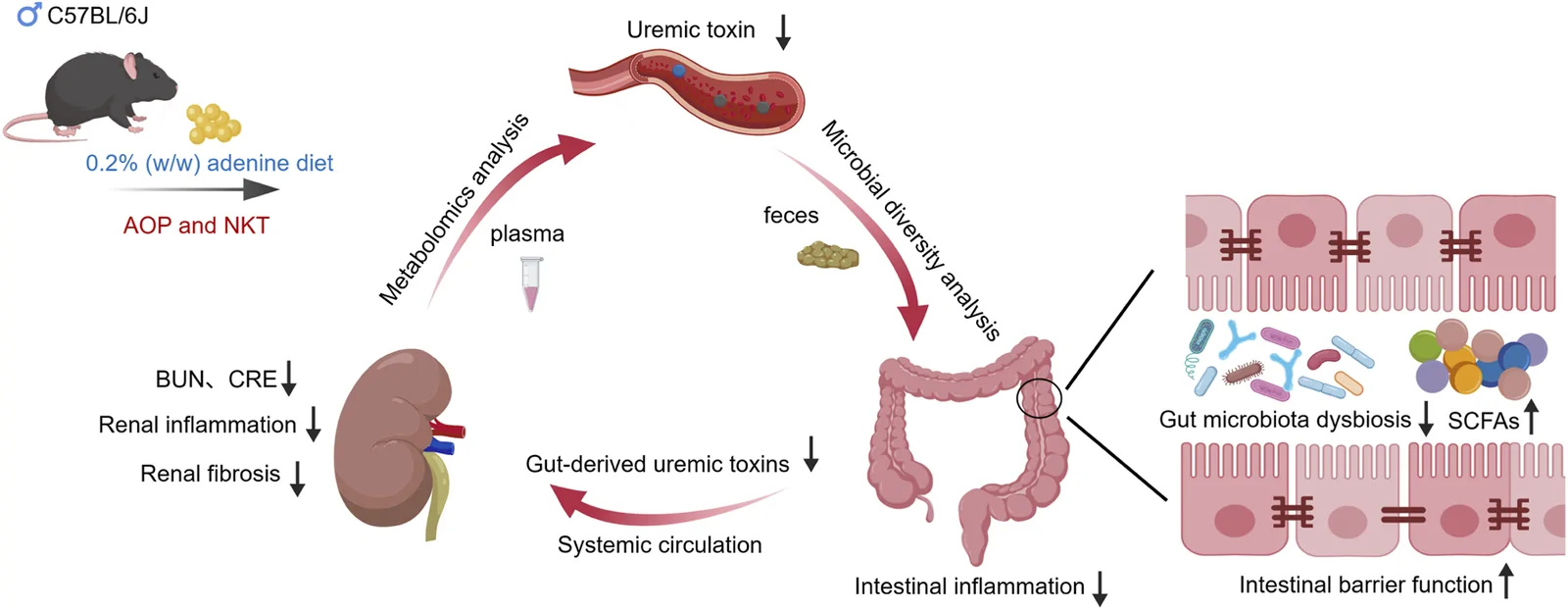

## 1 Introduction

Chronic kidney disease (CKD), with a reported global median prevalence of approximately 9.5%, has become a major public health challenge worldwide (Bello et al., 2024). It is characterized by progressive renal dysfunction and metabolic disturbances, with incidence increasing in parallel with population ageing, diabetes, hypertension, and elevated body mass index (GBD Chronic Kidney Disease Collaboration, 2020; Ruiz-Ortega et al., 2020). Early CKD is often asymptomatic, and progressive loss of renal function eventually leads to end-stage disease requiring dialysis or kidney transplantation (Kalyesubula and Luyckx, 2025). Globally, approximately 1.5 million deaths were attributed to CKD in 2021, with mortality continuing to rise (Deng L. et al., 2025). Current CKD management relies on dietary control, pharmacotherapy (e.g., RAAS and SGLT2 inhibitors), and renal replacement therapy (Adair and Bowden, 2020; Romagnani et al., 2025), yet limitations such as adverse effects, high costs, and resource constraints persist, underscoring the urgent need for novel interventions (Wong et al., 2024).

Increasing evidence underscores the pivotal role of the gut-kidney axis in CKD progression (Tao et al., 2024). Studies indicate that CKD markedly disrupts gut microbiota, resulting in substantial changes in its composition and function, as documented in both clinical cohorts and experimental models (Ren et al., 2020; Miao et al., 2024a). Microbial dysbiosis during disease states leads to multiple pathological consequences, including reduced production of beneficial metabolites, increased generation of gut-derived uremic toxins, and impaired intestinal barrier integrity. These changes promote systemic inflammation and oxidative stress, thereby accelerating renal injury (Chen et al., 2019; Meijers et al., 2019; Li X. -J. et al., 2024). Recent studies using joint metabolomics and gut microbiomics analyses have further revealed the association between gut microbial imbalance, toxin accumulation, and CKD progression (Wang et al., 2020). Notably, natural medicines and their bioactive metabolites have demonstrated antioxidant, anti-inflammatory, anti-fibrotic, and microbiota-modulating properties, suggesting anti-CKD potential through regulation of the gut-kidney axis (Shi et al., 2025).

*Alpinia oxyphylla* Miq. has been used for centuries in China as both a functional food and a traditional medicine (Zhao et al., 2016; Zhang et al., 2018). Studies have shown that *A. oxyphylla* fructus polysaccharide (AOP) exhibits various activities, including immunomodulatory, antioxidant, and anti-neuroinflammatory effects (Yang et al., 2020; Wang et al., 2024; Shi et al., 2020). Recent studies suggest that AOP exerts its biological activity, at least in part, through modulation of gut microbiota. AOP exerts pronounced regulatory effects on the intestinal microenvironment, including reshaping gut microbial composition, alleviating intestinal oxidative stress, and enhancing epithelial barrier integrity (Ma et al., 2023). Importantly, AOP has also been shown to alleviate renal inflammation and hyperuricemia by regulating metabolic pathways and promoting uric acid (UA) excretion through gut microbiota-dependent mechanisms (Ren et al., 2024). Nootkatone (NKT), the major sesquiterpenoid of *A. oxyphylla* fructus, exhibits antioxidant and anti-inflammatory properties. NKT demonstrates protective effects against renal disease in mouse models of carbon tetrachloride (CCl_4_) and unilateral ureteral obstruction (UUO) (Dai et al., 2023; Chen et al., 2021). However, NKT is characterized by poor aqueous solubility and low oral bioavailability, the latter of which has been shown to be further exacerbated in CKD models, severely limiting its application (Li et al., 2019). Emerging evidence indicates that polysaccharide-mediated remodeling of the intestinal microenvironment elevates the bioavailability and bioactivity of low-molecular-weight metabolites (Fu et al., 2020). However, investigations of *A. oxyphylla* fructus have primarily focused on crude extracts or individual metabolites, with limited exploration of potential interactions among its active metabolites. Given that both AOP and NKT are key bioactive metabolites with potential renoprotective properties, and considering the potential of polysaccharides to improve the bioavailability of low-molecular-weight metabolites, we hypothesized that their combination may exert greater renoprotective effects. The present study aimed to evaluate the individual and combined renoprotective effects of AOP and NKT in CKD, and to elucidate the underlying mechanisms through integrated metabolomics and gut microbiota analysis.

## 2 Materials and methods

### 2.1 Reagents and chemicals

The dried fruit of *A. oxyphylla* Miq. (Zingiberaceae; *Alpiniae oxyphyllae* fructus, Batch No. 210501) was supplied by LinShi ShengTai Pharmaceutical Co., Ltd. (Hainan, China) and authenticated by Professor Li Yonghui. A representative voucher specimen has been deposited at the Hainan Provincial Key Laboratory of R&D on Tropical Herbs, Hainan Medical University.

Nootkatone (Lot No. C13588822, purity 98%), propionic acid (Lot No. C15750961, purity ≥99.5%), butyric acid (Lot No. C17175080, purity 99%), isobutyric acid (Lot No. C16102498, purity 99%), valeric acid (Lot No. 17010258, purity ≥99.5%), isovaleric acid (Lot No. C17356485, purity 99%), crotonic acid (Lot No. C16902919, purity 98%) and trichloroacetic acid were acquired from Macklin Biochemical Co., Ltd. (Shanghai, China). Anhydrous ethanol, acetic acid (Lot No. B2311271, purity ≥99.5%), phosphoric acid, and sodium chloride (NaCl) were purchased from Xilong Scientific Co., Ltd. (Foshan, Guangdong). Acetonitrile and methanol (both HPLC-grade) were sourced from Thermo Fisher Scientific Co., Ltd. (Shanghai, China). Sodium hydroxide was supplied by Tianjin Damao Chemical Reagent Factory (Tianjin, China). Formic acid was purchased from Aladdin Biochemical Technology Co., Ltd. (Shanghai, China). All reagents listed above, unless otherwise specified, were of analytical grade.

### 2.2 Preparation of AOP

The preparation of AOP followed the protocol established in our previous work (Deng D. Z. et al., 2025), with key parameters summarized below and full structural characterization data provided in Supplementary Text S1. Briefly, *A. oxyphylla* fructus was crushed and sieved (60 mesh), immersed in 95% ethanol overnight, and then dried in an oven at 60 °C. The powder was extracted at 90 °C under reflux for 2 h with 0.1 M NaCl (1:20, w/v). The extract was concentrated before being precipitated overnight by the addition of a fourfold volume of anhydrous ethanol. Proteins were removed by the trichloroacetic acid method. Ethanol precipitation was performed again. The precipitate was reconstituted and dialyzed (molecular weight cutoff: 7 kDa, 72 h) with ultrapure water and lyophilized to yield AOP. The drug–extract ratio (DER) was 89:1 (w/w).

### 2.3 Animal experiment

Male C57BL/6J mice (5–6 weeks, 20 ± 2 g, SPF) were provided by Huachuang Xinnuo Pharmaceutical Technology Co., Ltd. (Jiangsu, China, Certificate No. B202407170117). The animals were housed under a 12 h light/dark cycle at 24 °C ± 2 °C and 50% ± 5% relative humidity. The animals had free access to food and water. All animal experimental procedures in this study were conducted in accordance with the guidelines for the care and use of laboratory animals, and were approved by the Ethics Committee of Hainan Medical University (Approval No. HYPLL-2026–001).

Following a 1-week acclimatization period, the mice were randomly assigned to five groups (n = 8 per group): the control group (CON), model group (MOD), AOP group (AOP), NKT group (NKT), and combination group (AOP-NKT). The CON group was fed a standard maintenance diet, while the other four groups were fed a 0.2% adenine diet for 6 weeks to induce CKD (Zaaba et al., 2023). The adenine diet was custom-prepared by Jiangsu Xietong Pharmaceutical Bio-Engineering Co., Ltd. (Nanjing, China). Compared with the CON group, the CKD model was considered established based on elevated blood urea nitrogen (BUN) and creatinine (CRE) levels, together with characteristic renal histopathological changes.

Model induction and drug administration were conducted simultaneously for 6 weeks. The doses of AOP (Ma et al., 2023) and NKT (Dai et al., 2023) were adopted from prior reports. Mice in the AOP, NKT, and AOP-NKT groups received AOP (300 mg/kg/day), NKT (10 mg/kg/day), or AOP-NKT (AOP 300 mg/kg/day and NKT 10 mg/kg/day). AOP was dissolved in ultrapure water. NKT was dispersed in ultrapure water and sonicated intermittently with vortexing to obtain a suspension. The suspension was freshly prepared immediately before administration to minimize sedimentation and ensure dosing consistency. Ultrapure water served as the vehicle for all groups. The CON and MOD groups received vehicle only. All mice were gavaged once daily with equal volumes standardized to body weight (0.125 mL per 10 g).

During the animal experiments, mouse body mass was monitored weekly, and daily records were kept of their diet and water intake. On the last day of the sixth week, fresh feces were collected and immediately stored in a −80 °C freezer. After fasting for 12 h, mice were anesthetized with isoflurane inhalation, blood was collected under anesthesia, and mice were euthanized by cervical dislocation. All procedures were conducted in accordance with the institutional guidelines for the care and use of laboratory animals. Plasma was then separated by centrifugation (4 °C, 3500 rpm, 15 min) and stored at −80 °C. Kidney and colon tissues, along with colonic contents, were then collected. Kidney weight and colon length were measured. For histopathological analysis, the left kidney and a 1–2 cm colon segment were fixed in 4% paraformaldehyde, while the remaining specimens were immediately transferred to a −80 °C freezer for subsequent experiments.

### 2.4 Biochemical analysis

The concentrations of plasma BUN and CRE were quantified with commercial assay kits purchased from the Nanjing Jiancheng Bioengineering Institute (Nanjing, China).

The kidney index was computed as follows: (kidney weight/body weight) × 100%.

### 2.5 Histological analysis

The tissues of kidney and colon were dehydrated, paraffin-embedded, and sectioned. Renal sections underwent H&E and Masson staining. Colon sections were stained with H&E and alcian blue-periodic acid Schiff (AB-PAS). The stained sections were examined by light microscopy.

### 2.6 Reverse transcription quantitative PCR (RT-qPCR) analysis

Total RNA from kidney or colon tissues was extracted using the Eastep® Super Total RNA Extraction Kit (Promega, Shanghai, China). cDNA was synthesized using the Hifair® III 1st Strand cDNA Synthesis SuperMix for qPCR kit (Yeasen, China). A 20 μL amplification reaction was prepared using Hieff® qPCR SYBR Green Master Mix (Low Rox Plus) kit (Yeasen, China) and run on the CFX96 Real-Time System (Bio-Rad). *β-actin* served as the reference gene. Data were analyzed using the 2^−ΔΔCT^ method and expressed relative to the control group. All primer sequences are listed in Supplementary Table S1.

### 2.7 Metabolomics analysis

Plasma sample (100 μL) was vortexed with methanol/acetonitrile (300 μL) (1:1, v/v) extraction solvent and maintained at −20 °C for 1 h. The supernatant was separated by centrifugation and evaporated under a gentle air stream. The sample residues were reconstituted by vortexing with pre-cooled 50% acetonitrile, and the resulting supernatant was collected following centrifugation for subsequent analysis. Metabolomic analysis was performed using ultra-high performance liquid chromatography coupled with quadrupole time-of-flight mass spectrometry (AB Sciex Triple-TOF 4600, United States of America). Chromatographic and mass-spectrometric parameters were detailed in Supplementary Text S2.

Data processing and analysis were performed as previously described (Xu et al., 2022). Briefly, raw data were preprocessed using MarkerView1.3.1 (AB Sciex, United States of America). Processed datasets were imported into SIMCA 14.1.0 (Umetrics, Sweden) for multivariate statistical modelling. Secondary mass spectrometry information was obtained using PeakView 1.2.1 (AB Sciex, United States of America). Metabolites were annotated against HMDB (https://hmdb.ca/), PubChem (https://pubchem.ncbi.nlm.nih.gov), and the KEGG (https://www.kegg.jp/) database. To visualize the data, we employed the tools of OmicStudio platform (https://www.omicstudio.cn/tool). Finally, pathway enrichment was performed with MetaboAnalyst 6.0 platform (https://www.metaboanalyst.ca/).

### 2.8 Gut microbiota analysis

Total microbial genomic DNA was extracted from mouse fecal samples using the FastPure Feces DNA Isolation Kit (Majorbio Bio-pharm Technology Co., Ltd., Shanghai, China). DNA quality was assessed by agarose gel electrophoresis, and its concentration was quantified by a NanoDrop2000 spectrophotometer (Thermo Fisher Scientific, United States of America). PCR amplification of the V3-V4 hypervariable region of the 16S rRNA gene was carried out with forward primer 338F (5′-ACT​CCT​ACG​GGA​GGA​GGC​AG-3′) and reverse primer 806R (5′-GGACTACHVGGGTWTCTAAT-3′). Amplicons were excised from 2% agarose gel, purified with the PCR Clean-Up Kit (YuHua, China), and quantified using a Qubit 4.0 fluorometer (Thermo Fisher Scientific, United States of America). The NEXTFLEX Rapid DNA-Seq Kit (Bioo Scientific, United States of America) was used to perform library preparation. The Illumina MiSeq PE300 system was utilized to conduct all high-throughput sequencing in this study. All bioinformatic analyses were completed with the MajorBio Cloud platform (https://cloud.majorbio.com).

### 2.9 Short-chain fatty acids (SCFAs) determination

Mouse fecal sample was mixed with ultrapure water (1:5, w/v, containing 100 μg/mL internal standard crotonic acid) and homogenized using a tissue homogenizer (Wuhan Servicebio Technology Co., Ltd.). After a 15-min centrifugation at 4 °C and 8000 rpm, the clarified supernatants were moved to fresh centrifuge tubes and adjusted to pH 2.0–3.0 with 10% phosphoric acid. After acidifying the sample on ice for 1 h, the sample was combined with an equal volume of ethyl acetate and vortexed for 5 min. Finally, the organic phase obtained after centrifugation (4 °C, 13,000 rpm, 15 min) was used for analysis.

Gas chromatography (GC) analysis was conducted using a Thermo Fisher Scientific Trace 1300 gas chromatography system (Thermo Fisher Scientific, United States of America) equipped with a flame-ionization detector (FID) to quantify the concentrations of SCFAs, including acetic acid, propionic acid, isobutyric acid, butyric acid, isovaleric acid and valeric acid. Separation was achieved on a ZB-WAXplus capillary column (30 m × I.D. = 0.53 mm × df = 1.00 μm, Phenomenex, United States of America). The equipment was operated in splitless mode with an injection volume of 2 μL, and high-purity nitrogen was used as the carrier gas at a constant flow of 2 mL/min. The injection port temperature was maintained at 280 °C, while the FID temperature was set to 300 °C. The gases were supplied at the following flow rates: hydrogen, 35 mL/min; high-purity nitrogen, 40 mL/min; and synthetic air, 350 mL/min. The column oven temperature started at 90 °C (2-min hold), then rose to 150 °C at 5 °C/min (5-min hold), followed by an increase to 170 °C at 4 °C/min (3-min hold), with a final ramp to 200 °C at 10 °C/min (3-min hold). Data acquisition and analysis were conducted using Chromeleon CDS software (Thermo Fisher Scientific, United States of America).

### 2.10 Immunohistochemical (IHC) analysis

Colon tissue sections embedded in paraffin were first dewaxed and rehydrated. Antigen retrieval was subsequently performed using EDTA buffer. The sections were exposed to a 3% H_2_O_2_ solution to quench endogenous peroxidase activity, followed by blocking with 3% BSA. After removal of the blocking solution, the sections were probed with primary antibodies against zonula occludens-1 (ZO-1) (1:200, Servicebio, GB15195), occludin (1:500, Servicebio, GB111401), and claudin-1 (1:500, Servicebio, GB15032) overnight at 4 °C. On the following day, at room temperature, the sections were exposed to the corresponding secondary antibodies for 50 min, followed by visualization using DAB and hematoxylin counterstaining of nuclei. Finally, the sections were dehydrated and mounted. Representative areas of immunostained tissue were captured using a light microscope (E100, Nikon Instruments Ltd., Japan). Immunohistochemical images were quantified using the AI-based scoring software AIpathwell (Servicebio, Wuhan, China), and the H-score was used for subsequent analysis (Li Y. et al., 2024).

### 2.11 Statistical analysis

Statistical analysis and graphing were performed using GraphPad Prism (version 10.4.0), with results expressed as the mean ± standard deviation (SD). One-way or two-way analysis of variance (ANOVA) was used for multi-group comparisons as appropriate, following assessment of normality and homogeneity of variance. Tukey’s *post hoc* test was applied for multiple comparisons when parametric assumptions were met. When these assumptions were not satisfied, the Kruskal–Wallis test followed by Dunn’s *post hoc* test was performed. A p-value < 0.05 was considered statistically significant. Graphical abstract was created with BioGDP.com.

## 3 Results

### 3.1 Effects of AOP-NKT on physiological indices in CKD mice

**FIGURE 1:**
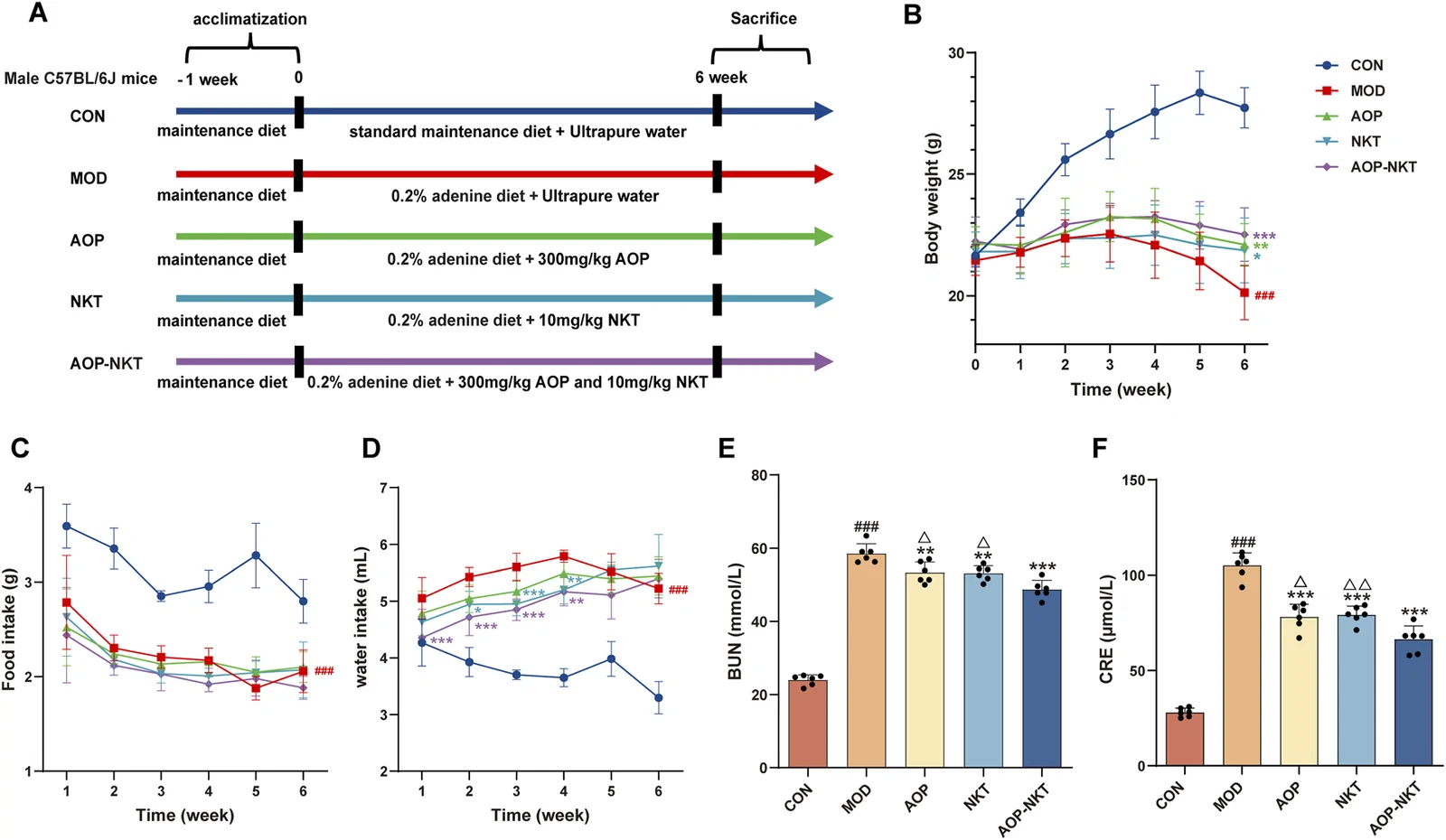
Effects of AOP, NKT, and AOP-NKT on physiological indices in adenine-induced CKD mice. **(A)** Experimental design. **(B)** Body weight curves. **(C)** Food intake. **(D)** Water intake. **(E)** Plasma urea nitrogen (BUN). **(F)** Plasma creatinine (CRE). Data are presented as mean ± SD (n = 6). #p < 0.05, ##p < 0.01, ###p < 0.001 vs. CON group; *p < 0.05, **p < 0.01, ***p < 0.001 vs. MOD group; △ p < 0.05, △△ p < 0.01, △△△ p < 0.001 vs. AOP-NKT group.

The design of animal experiments was shown in Figure 1A. Relative to the CON group, mice in the MOD group exhibited significant reductions in body weight and food intake (both p < 0.001). Water intake in MOD mice exhibited a biphasic change, characterized by an initial increase followed by a subsequent decrease (p < 0.001). The latter decrease in water intake might be caused by the continuous deterioration of the disease. Administration of AOP, NKT alone, or both in combination significantly ameliorated weight loss. Notably, the AOP-NKT group showed significant inhibition of both weight loss and initial increase in water intake in CKD mice (p < 0.001). All adenine-diet groups showed similar food intake (Figures 1B–D). In addition, BUN and CRE levels were significantly higher in the MOD group versus the CON group (p < 0.001), indicating renal dysfunction and supporting the establishment of the CKD mouse model. The AOP-NKT group exhibited more significant reductions in both BUN and CRE levels compared with the AOP group or NKT group alone (Figures 1E,F).

### 3.2 AOP-NKT ameliorates renal inflammation and fibrosis in CKD mice

**FIGURE 2:**
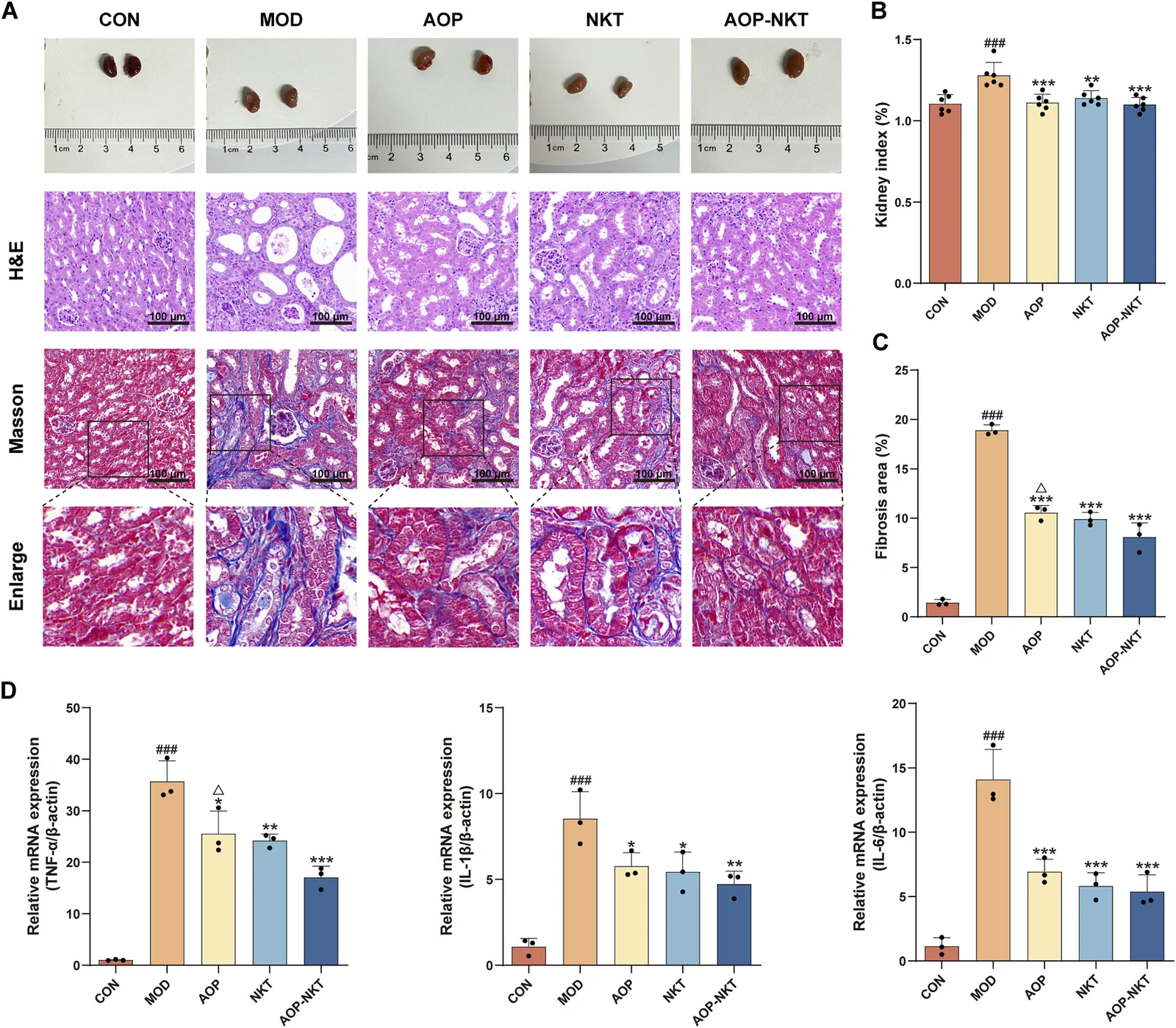
AOP-NKT restores renal architecture and alleviates inflammation in CKD mice. **(A)** Representative gross renal morphology and histological sections (H&E and Masson). Scale bar = 100 μm. **(B)** Kidney index. **(C)** Statistical analysis of fibrosis percentage in renal tissue sections. **(D)** Renal mRNA levels of TNF-α, IL-1β, and IL-6. Data are expressed as mean ± SD (n = 6 for B and n = 3 for **(C and D)**). #p < 0.05, ##p < 0.01, ###p < 0.001 vs. CON group; *p < 0.05, **p < 0.01, ***p < 0.001 vs. MOD group; △ p < 0.05, △△ p < 0.01, △△△ p < 0.001 vs. AOP-NKT group.

Renal specimens harvested from MOD animals, unlike CON counterparts, appeared whitish and were characterized by protrusions resembling small nodules on the renal surface; concurrently, the kidney index in this group was significantly elevated (p < 0.001). H&E staining of sections from CKD mice revealed glomerular atrophy, obvious dilatation of renal tubules, irregular shape of tubular lumen, accompanied by marked interstitial infiltration of inflammatory cells within the renal tissue. After drug intervention, the renal structure was markedly restored, and the degree of inflammatory cell infiltration and tubular damage was significantly reduced. Quantitative analysis of collagen deposition area on Masson-stained sections showed that the fibrotic areas were markedly reduced in the AOP group (10.56% ± 0.72%), the NKT group (9.90% ± 0.68%), and the AOP-NKT group (8.08% ± 1.40%), compared with the MOD group (18.91% ± 0.54%) (Figures 2A–C). As shown in Figure 2D, RT-qPCR analysis of inflammation-related gene expression in kidney tissue revealed that the MOD group exhibited substantially higher mRNA levels of TNF-α, IL-1β, and IL-6 compared with the CON group (p < 0.001). The pharmacological interventions significantly inhibited the upregulation of those genes, especially in the AOP-NKT group. Notably, the AOP-NKT group exhibited greater improvements in renal structure, fibrosis, and inflammation than either AOP or NKT alone.

### 3.3 AOP-NKT ameliorates the pathological damage of the colon in CKD mice

**FIGURE 3:**
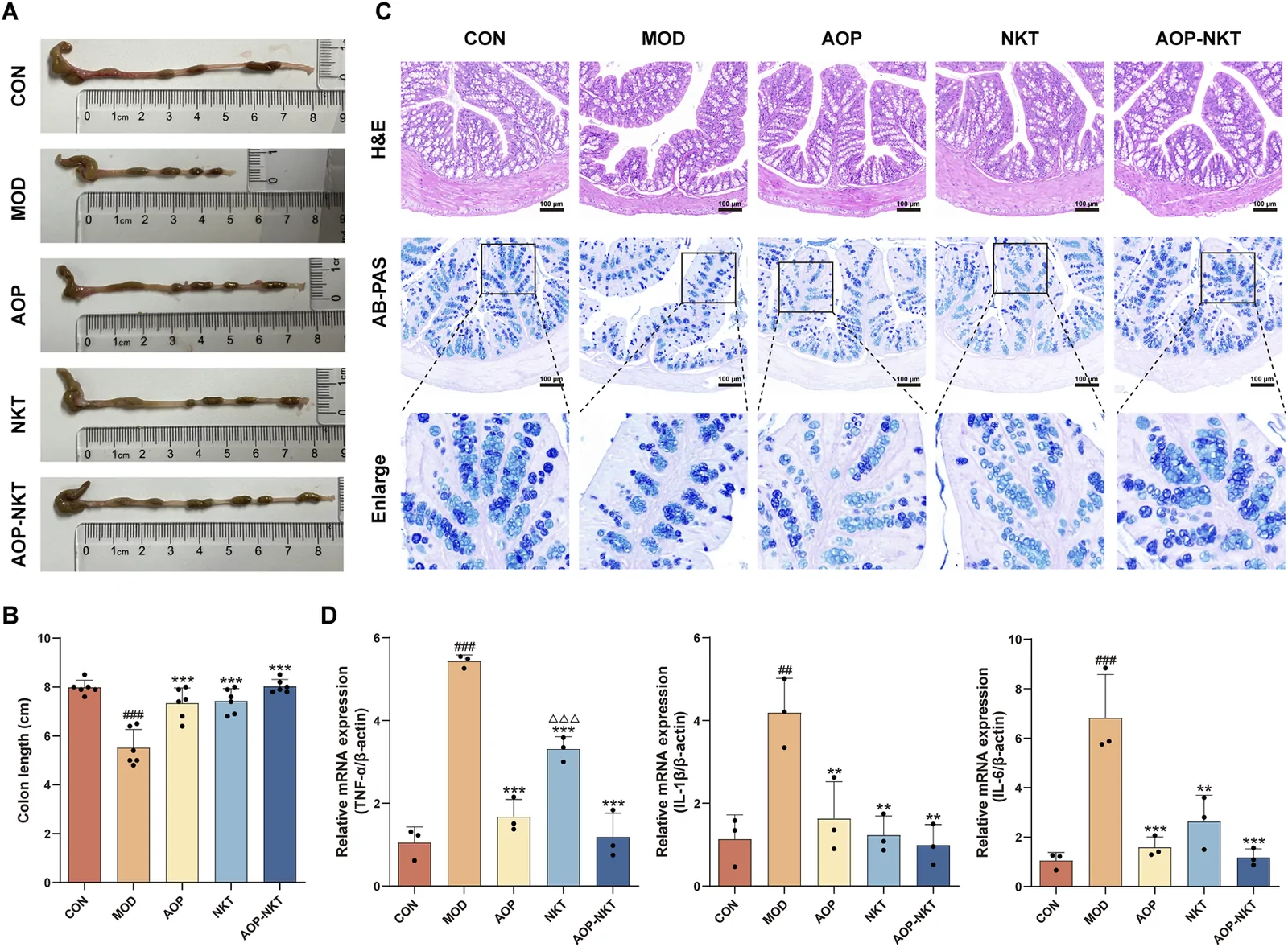
AOP-NKT alleviates pathological damage in the colon of CKD mice. **(A)** Representative gross colonic morphology. **(B)** Colonic length. **(C)** Representative colonic histological sections (H&E and AB-PAS). Scale bar = 100 μm. **(D)** Colonic mRNA levels of TNF-α, IL-1β, and IL-6. Data are expressed as mean ± SD (n = 6 for B and n = 3 for D). #p < 0.05, ##p < 0.01, ###p < 0.001 vs. CON group; *p < 0.05, **p < 0.01, ***p < 0.001 vs. MOD group; △ p < 0.05, △△ p < 0.01, △△△ p < 0.001 vs. AOP-NKT group.

We subsequently assessed the pathological changes of the colon. Colons from MOD mice were significantly shorter than those from CON mice (p < 0.001) (Figures 3A,B). H&E staining of the colon revealed goblet cell dissolution, inflammatory cell infiltration, crypt atrophy, and submucosal oedema in the colonic mucosal layer of CKD mice. AB-PAS staining revealed that the mucus layer of the colon in CKD mice was severely damaged, with a significant reduction of goblet cells and mucin-rich stained areas, especially near the intestinal lumen. Following administration, significant improvements were observed in colon shortening and histopathological damage, with the AOP-NKT group exhibiting the most pronounced preservation of the mucus layer (Figure 3C). As demonstrated in Figure 3D, the MOD group exhibited pronounced upregulation of TNF-α, IL-1β, and IL-6 mRNA in colonic tissues. AOP and NKT administration significantly alleviated the colonic inflammatory state in CKD mice, particularly in the AOP-NKT group.

### 3.4 AOP-NKT ameliorates plasma metabolite imbalance in CKD mice

**FIGURE 4:**
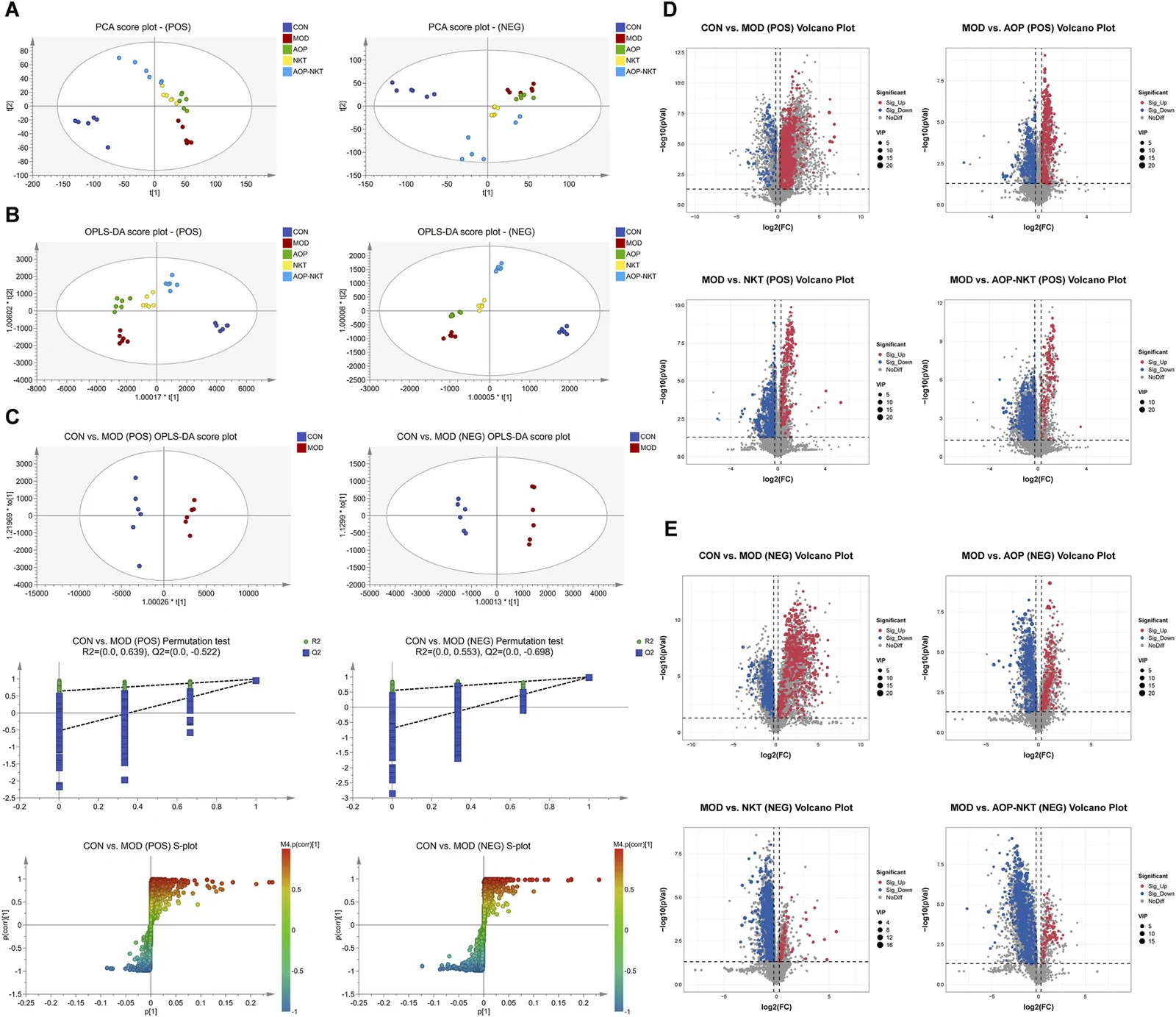
Multivariate analysis of plasma metabolomes distinguishes experimental groups. **(A)** PCA score plots in positive and negative ion modes. **(B)** OPLS-DA score plots in positive and negative ion modes. **(C)** OPLS-DA score plots, 200-permutation test plots and S-plots of the CON and MOD groups in positive and negative ion modes. **(D)** Volcano plots between groups in positive ion mode. **(E)** Volcano plots between groups in negative ion mode.

Metabolic imbalance is a hallmark of CKD progression. We used untargeted metabolomics to compare changes in plasma metabolites among different experimental groups of mice. The total ion chromatograms (TICs) of the QC samples and representative experimental groups in positive and negative ion modes were shown in Supplementary Figure S1. The good overlap of QC samples demonstrated excellent system stability and reproducibility throughout the analysis. After data preprocessing, 9588 and 7875 feature peaks were extracted under positive and negative ion modes, respectively. Marked segregation of the CON and MOD groups in plasma metabolic profiles was revealed by principal component analysis (PCA) score plots (Figure 4A), indicative of substantial metabolic perturbations in CKD mice. The Orthogonal partial least squares discriminant analysis (OPLS-DA) score plot (Figure 4B) illustrated strong intra-group aggregation and clear inter-group separation. The AOP, NKT, and AOP-NKT groups tended to cluster closer the CON group, especially the AOP-NKT group. The model validation was conducted using 200 permutation tests (CON vs. MOD) (Figure 4C). The original model’s R^2^ and Q^2^ values exceeded those of the random permutation distribution, with a negative regression intercept for Q^2^ also observed, indicating no overfitting and confirming the reliability of the model in this analysis. In addition, variables at the distal ends of the S-plot contributed the most to group discrimination and were prioritized as candidate differential metabolites for subsequent analysis.

The study’s screening criteria for differentially expressed plasma metabolites included VIP > 1, p < 0.05, and FC > 1.2 or < 0.8. In positive ion mode, the volcano plot (Figure 4D) showed that the MOD group had 802 upregulated and 104 downregulated differential feature peaks versus the CON group. Relative to the MOD group, the AOP, NKT, and AOP-NKT groups had 290, 188, and 121 upregulated differential feature peaks, and 278, 398, and 565 downregulated differential feature peaks, respectively. In negative ion mode (Figure 4E), the MOD group had 499 upregulated and 506 downregulated differential feature peaks versus the CON group. Relative to the MOD group, the AOP, NKT, and AOP-NKT groups had 198, 92, and 68 upregulated differential feature peaks, and 407, 778, and 747 downregulated differential feature peaks, respectively.

**FIGURE 5:**
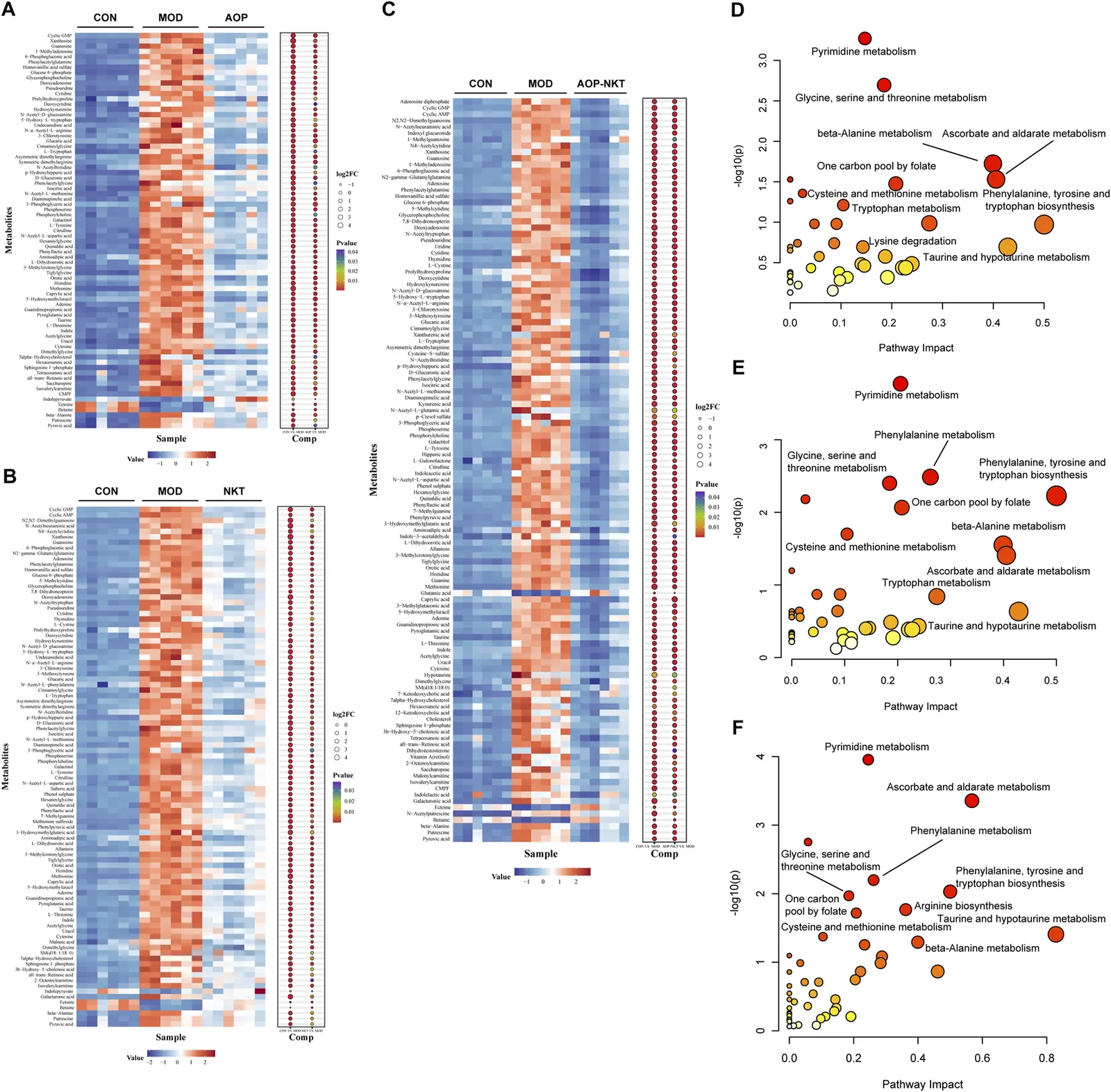
Heatmap of differential metabolites and KEGG metabolic pathway enrichment analysis. **(A)** Heatmap of CON, MOD and AOP groups. **(B)** Heatmap of CON, MOD and NKT groups. **(C)** Heatmap of CON, MOD and AOP-NKT groups. **(D–F)** KEGG pathway enrichment analysis for AOP **(D)**, NKT **(E)** and AOP-NKT **(F)** groups, respectively.

Following database matching and identification, the CKD-related metabolite alterations reversed by each intervention were visualized in heat maps (Figures 5A–C). The AOP, NKT, and AOP-NKT interventions significantly regulated 75, 95, and 118 metabolites, respectively (Supplementary Tables S2–S4). The MetaboAnalyst 6.0 website was used to perform enrichment analysis related to metabolite disease characteristics (for uremia, kidney disease, and chronic renal failure). The AOP, NKT, and AOP-NKT groups exhibited 16, 17, and 25 kidney disease-related metabolites, respectively. Nine metabolites were uniquely altered in the AOP-NKT group compared with the NKT groups, including p-cresol sulfate, indoleacetic acid, hippuric acid, 3-carboxy-4-methyl-5-propyl-2-furanpropionic acid (CMPF), indoxyl glucuronide, kynurenic acid, uridine, 1-methyladenosine, and 1-methylguanosine. Notably, 1-methyladenosine and CMPF were also identified as differential metabolites in the AOP group, suggesting that the regulatory effects of the combined intervention on these two metabolites may be primarily attributable to AOP intervention. By contrast, the remaining seven metabolites were uniquely altered in the AOP-NKT combination group, indicating that co-administration of AOP and NKT may broaden the metabolic regulatory spectrum, particularly by enhancing the clearance of specific uremic toxins.

The online MetaboAnalyst 6.0 platform was utilized to conduct KEGG pathway enrichment analysis on the substantially altered metabolites among experimental groups (Figures 5D–F). With a pathway impact value > 0.1, 18, 19, and 21 pathways were identified in the AOP, NKT, and AOP-NKT groups, respectively. Key metabolic pathway information was obtained by combining FDR values and hit counts. Pyrimidine metabolism was the most significantly enriched pathway in the AOP, NKT, and AOP-NKT groups (AOP: FDR = 0.043, Hits = 6; NKT: FDR = 0.014, Hits = 7; AOP-NKT: FDR = 0.0089, Hits = 8). Notably, glycine-serine and threonine metabolism pathway was also generally enriched in these three groups. Relative to the AOP, the NKT and AOP-NKT groups exhibited a more pronounced enrichment trend for phenylalanine-tyrosine and tryptophan biosynthesis, phenylalanine metabolism, glycine-serine and threonine metabolism as well as the one-carbon pool by folate. Furthermore, compared with the AOP and NKT groups, the AOP-NKT group exhibited broader regulatory effects, accompanied by enrichment of more metabolites involved in purine metabolism, ascorbate and aldarate metabolism, and arginine biosynthesis pathways.

### 3.5 AOP-NKT remodels gut microbiota structure in CKD mice

**FIGURE 6:**
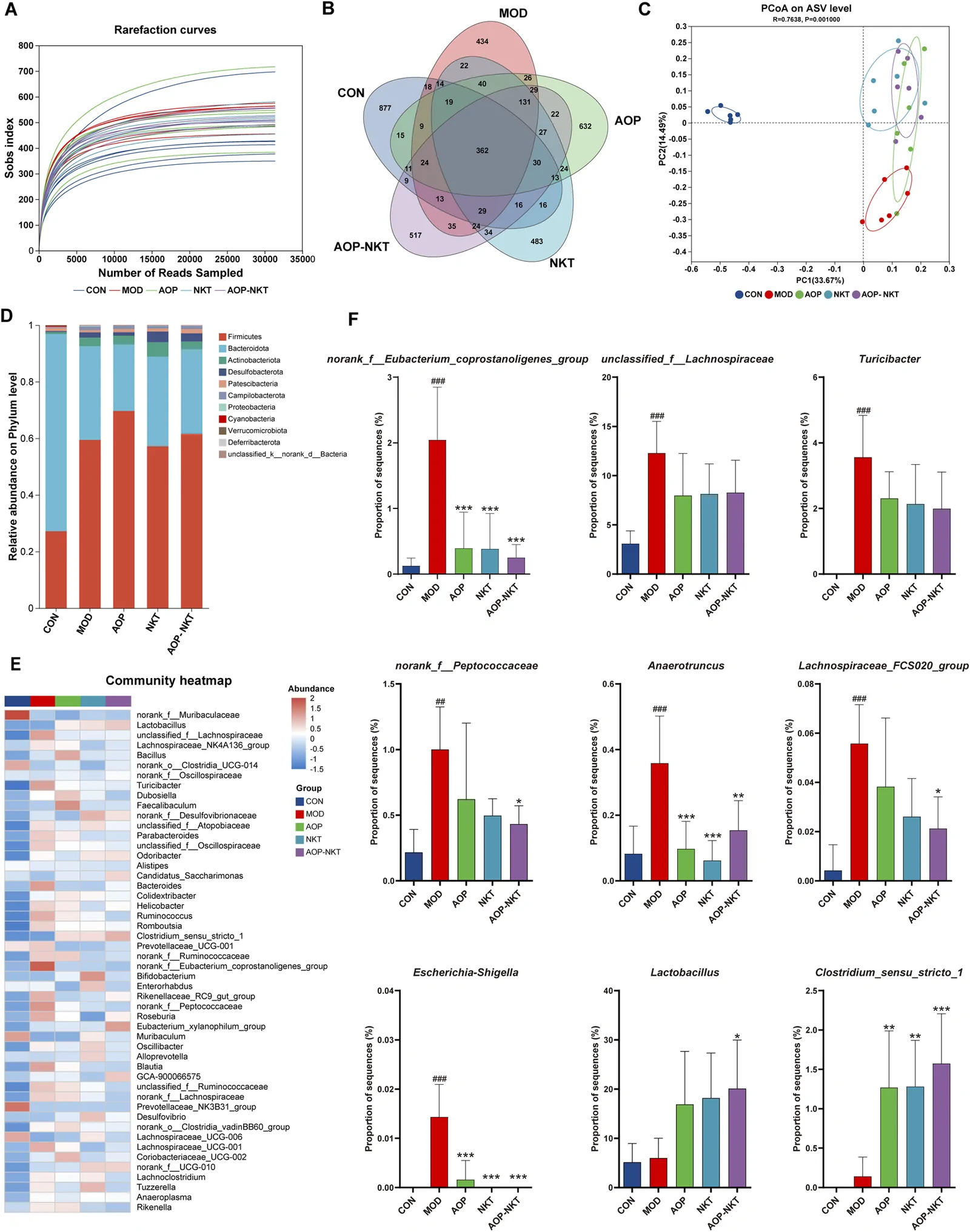
AOP-NKT improves gut microbiota dysbiosis in CKD mice. **(A)** Rarefaction curves (Sobs index). **(B)** Venn diagram of ASV levels. **(C)** PCoA on ASV level. **(D)** Phylum-level abundance. **(E)** Heatmap of top 50 genera. **(F)** Relative abundance of representative differential genera. Data are expressed as mean ± SD (n = 6). #p < 0.05, ##p < 0.01, ###p < 0.001 vs. CON group; *p < 0.05, **p < 0.01, ***p < 0.001 vs. MOD group.

To investigate the role of the gut microbiota in CKD progression and the effects of AOP, NKT, and their combined administration on microbial communities, gut microbiota analysis was performed on fecal specimens from mice. The raw sequencing reads were processed using the DADA2 algorithm within the QIIME2 environment to perform denoising, thereby yielding high-resolution amplicon sequence variants (ASVs). The sobs-indexed rarefaction curves at the ASV level tended to flatten, indicating sufficient sequencing data depth and reliable experimental results (Figure 6A). The Venn analysis revealed 362 overlapping ASVs common to all five groups. The MOD group had the fewest unique ASVs (434), while the AOP, NKT, and AOP-NKT groups had 632, 483, and 517 unique ASVs, respectively (Figure 6B).

Alpha diversity analysis provided insights into species richness and diversity within the community (Supplementary Figures S2A–D). The Ace, Chao, and Shannon indices of alpha diversity revealed a rising trend in the MOD group when compared with the CON group, whereas the Simpson index showed a decreasing trend. Following drug administration, the alpha diversity indices were slightly restored, but the differences were not statistically significant. Subsequent beta diversity analysis via principal coordinates analysis (PCoA) of Bray-Curtis distances at the ASV level demonstrated marked divergence in microbial community structure across experimental groups (R = 0.7638, P = 0.001). The CON and MOD groups showed significant separation along the PC1 axis (33.67%), indicating substantial remodeling of the intestinal microbiota in CKD mice. In addition, the AOP, NKT, and AOP-NKT groups showed considerable separation from the MOD group along the PC2 axis (14.49%). Meanwhile, it was observed that the AOP-NKT group partially overlapped with both the NKT group and the AOP group, suggesting that both AOP and NKT contribute to the gut microbial structure characteristics in the AOP-NKT group (Figure 6C).

We next examined the structural alterations of the gut microbiota. Taxonomic profiling at the phylum level revealed that Firmicutes and Bacteroidota predominated, exhibiting the highest relative abundances across all experimental groups. The MOD group exhibited significantly elevated relative abundances of Firmicutes, Actinobacteriota, Campilobacterota, and Proteobacteria compared with CON, concomitant with substantially reduced relative abundance of Bacteroidota (Figure 6D; Supplementary Figure S2E). The AOP and AOP-NKT interventions significantly reduced the abundance of Proteobacteria relative to MOD.

Genus-level profiling through heatmap visualization of the top 50 genera by relative abundance revealed significant restructuring of the microbial community in the MOD group in comparison to the CON group (Figure 6E). Relative to the MOD group, all three drug intervention groups partially restored the relative abundance of *unclassified_f__*Lachnospiraceae, *Turicibacter*, *Ruminococcus*, *Blautia*, and *Muribaculum*. These interventions also significantly reversed the relative abundance of *norank_f__Eubacterium_coprostanoligenes_group*, *Anaerotruncus*, *UCG-009*, *Eubacterium_nodatum_group* and *Escherichia-Shigella*. Furthermore, the AOP-NKT group considerably reduced the relative abundance of *norank_f__*Peptococcaceae and Lachnospiraceae*_FCS020_group* (p < 0.05 for both), whereas it markedly enhanced the levels of *Lactobacillus* (p < 0.05). The abundance of *Clostridium_sensu_stricto_1* was significantly elevated in the AOP, NKT, and AOP-NKT groups compared with the MOD group (Figure 6F; Supplementary Figure S2F). The drug interventions partially alleviated the gut microbiota dysbiosis observed in mice with CKD.

**FIGURE 7:**
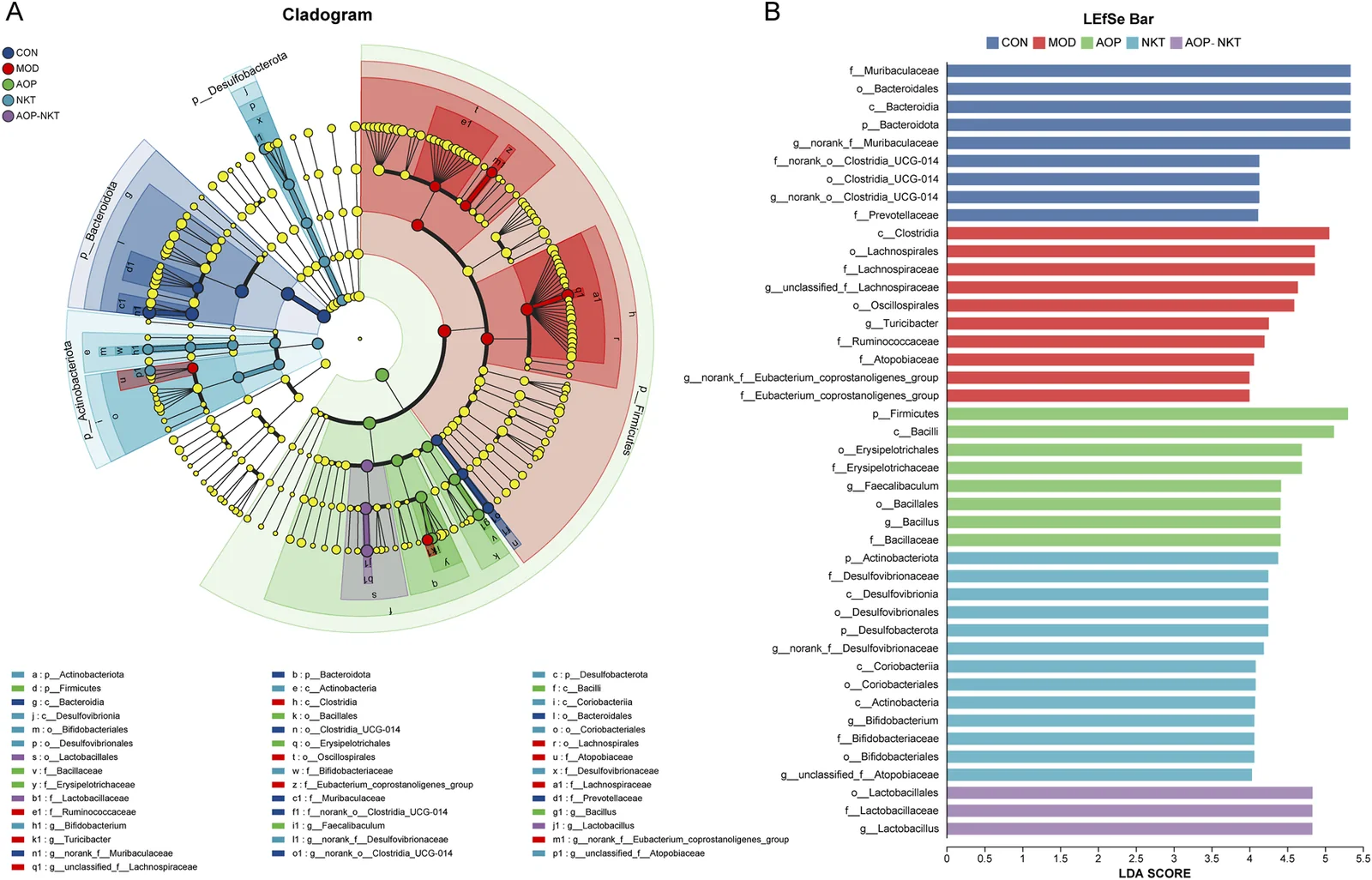
LEfSe analysis (LDA > 4, p < 0.05). **(A)** Cladogram of differentially abundant taxa. **(B)** LDA score plot.

To screen for discriminative taxonomic features among diverse phylogenetic levels, we employed Linear discriminant analysis effect size (LEfSe). The magnitude of differential enrichment was subsequently estimated using LDA (linear discriminant analysis) scores. As Figures 7A,B illustrates, taxa with LDA scores > 4 were identified as 43 differential taxonomic units at the phylum, class, order, family and genus levels. At the phylum level, Bacteroidota characterized the CON group, whereas taxa driving intergroup differences in the MOD group were predominantly affiliated with the phylum Firmicutes (spanning class to genus levels). At the genus level, *norank_f__Muribaculaceae* and *norank_o__Clostridia_UCG-014* were characteristic of the CON group, while *unclassified_f__* *Lachnospiraceae*, *Turicibacter* and *norank_f__Eubacterium_coprostanoligenes_group* were representative of the MOD group. Furthermore, the AOP group harbored *Faecalibaculum* and *Bacillus*. Meanwhile, the NKT group exhibited enrichment of *norank_f__* *Desulfovibrionaceae* *, Bifidobacterium* and *unclassified_f__Atopobiaceae*, whereas *Lactobacillus* emerged as the signature genus in the AOP-NKT group. These genera were characteristic of their respective groups and served as signature taxa.

### 3.6 AOP-NKT prevents the decline in intestinal SCFA levels and maintains intestinal barrier function in CKD mice

**FIGURE 8:**
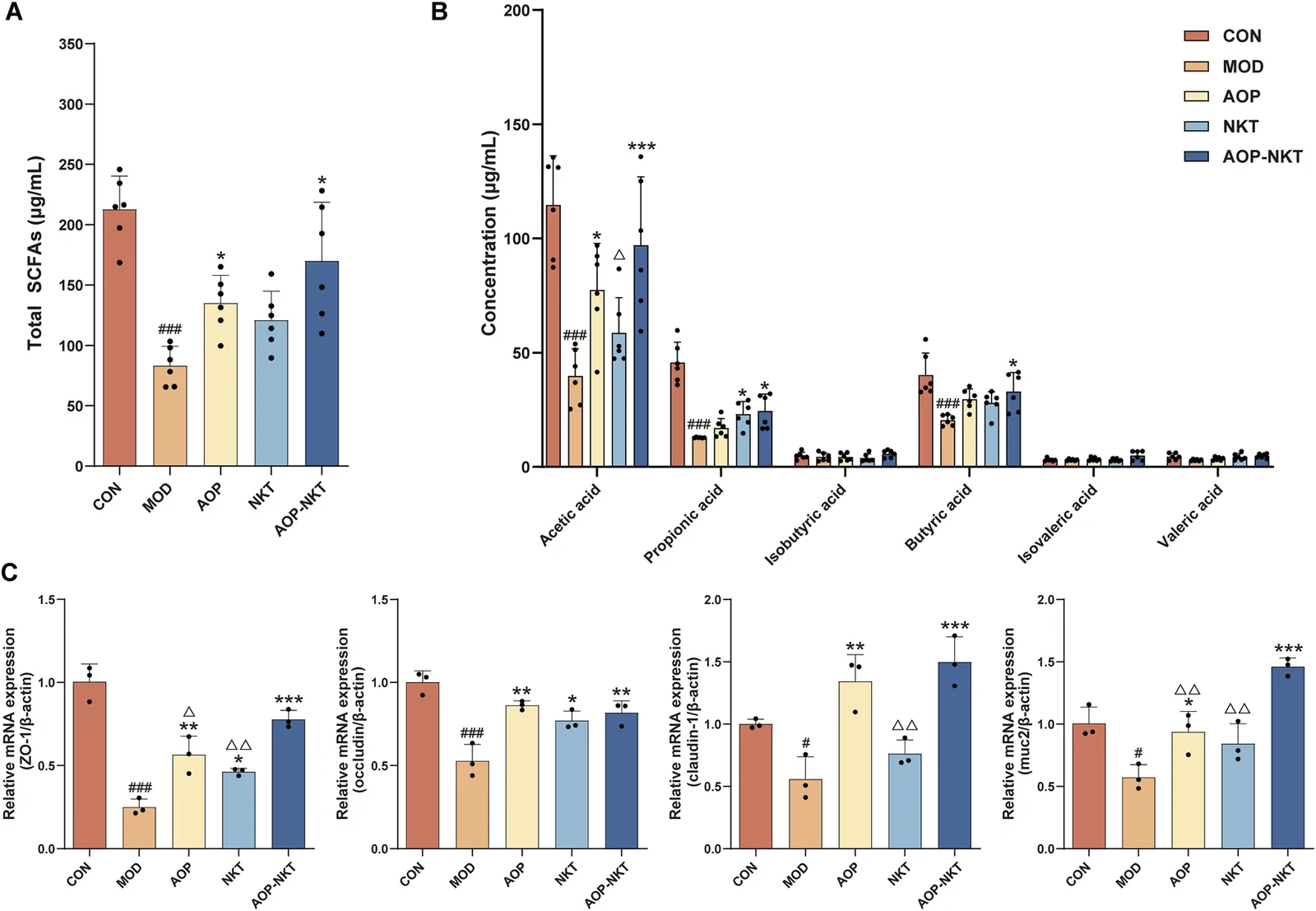
AOP-NKT increases intestinal SCFA levels and intestinal barrier-related mRNA expression in CKD mice. **(A)** Total SCFAs. **(B)** Individual SCFA concentrations. **(C)** Colonic mRNA levels of ZO-1, occludin, claudin-1 and muc2. Data are expressed as mean ± SD (n = 6 for A and B and n = 3 for C). #p < 0.05, ##p < 0.01, ###p < 0.001 vs. CON group; *p < 0.05, **p < 0.01, ***p < 0.001 vs. MOD group; △ p < 0.05, △△ p < 0.01, △△△ p < 0.001 vs. AOP-NKT group.

As pivotal metabolic products generated by the intestinal microbiota, SCFAs are essential for maintaining host-gut interactions. The SCFA concentrations in mouse feces were quantified by GC. Total SCFAs, acetic acid, propionic acid, and butyric acid levels were significantly lower in the MOD mouse feces relative to those in CON mice (p < 0.001), whereas valeric acid exhibited a declining pattern without statistical significance. The drug interventions prevented the decrease in SCFA levels in CKD mice, especially in the AOP-NKT group. In contrast to the MOD group, acetic acid in both AOP (p < 0.05) and AOP-NKT (p < 0.001) groups was markedly increased, and an increasing trend was observed in the NKT group. Propionic acid in both NKT and AOP-NKT groups exhibited a significant rise (p < 0.05). Butyric acid concentrations increased in the AOP, NKT, and AOP-NKT groups, most markedly in the AOP-NKT group (p < 0.05) (Figures 8A,B).

**FIGURE 9:**
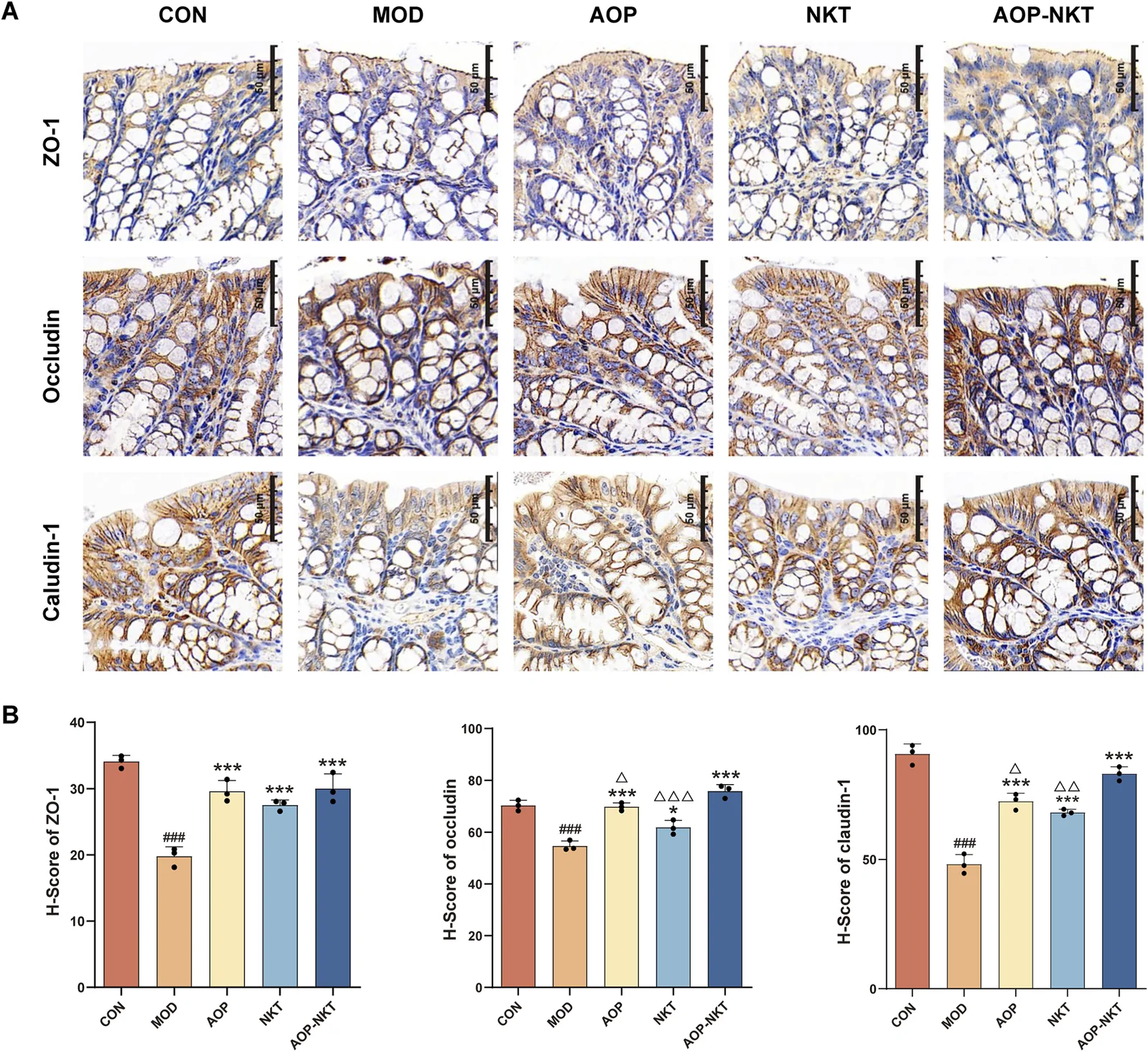
AOP-NKT enhances intestinal barrier function in CKD mice. **(A)** Representative IHC images of ZO-1, occludin, and claudin-1 in colon tissue. Scale bar = 50 μm. **(B)** Quantitative IHC (H-Score) for ZO-1, occludin, and claudin-1. Data are expressed as mean ± SD (n = 3 for B). #p < 0.05, ##p < 0.01, ###p < 0.001 vs. CON group; *p < 0.05, **p < 0.01, ***p < 0.001 vs. MOD group; △ p < 0.05, △△ p < 0.01, △△△ p < 0.001 vs. AOP-NKT group.

Intestinal barrier function and SCFA levels are inextricably intertwined, and we further investigated how drug interventions modulate colonic barrier function in CKD mice. Compared with the CON group, intestinal barrier-related gene expression (ZO-1, occludin, claudin-1, and muc2) was significantly downregulated in CKD mice (Figure 8C), and drug interventions significantly inhibited this downregulation. Consistent with the mRNA expression results, immunohistochemical (IHC) staining and quantitative analysis of H-Score statistics also demonstrated that drug interventions significantly prevented the decrease in expression of intestinal barrier-related proteins ZO-1, occludin, and claudin-1 in CKD mice, especially in the AOP-NKT group (p < 0.001) (Figures 9A,B).

### 3.7 Correlation analysis among gut microbiota, metabolites, and renal function parameters

**FIGURE 10:**
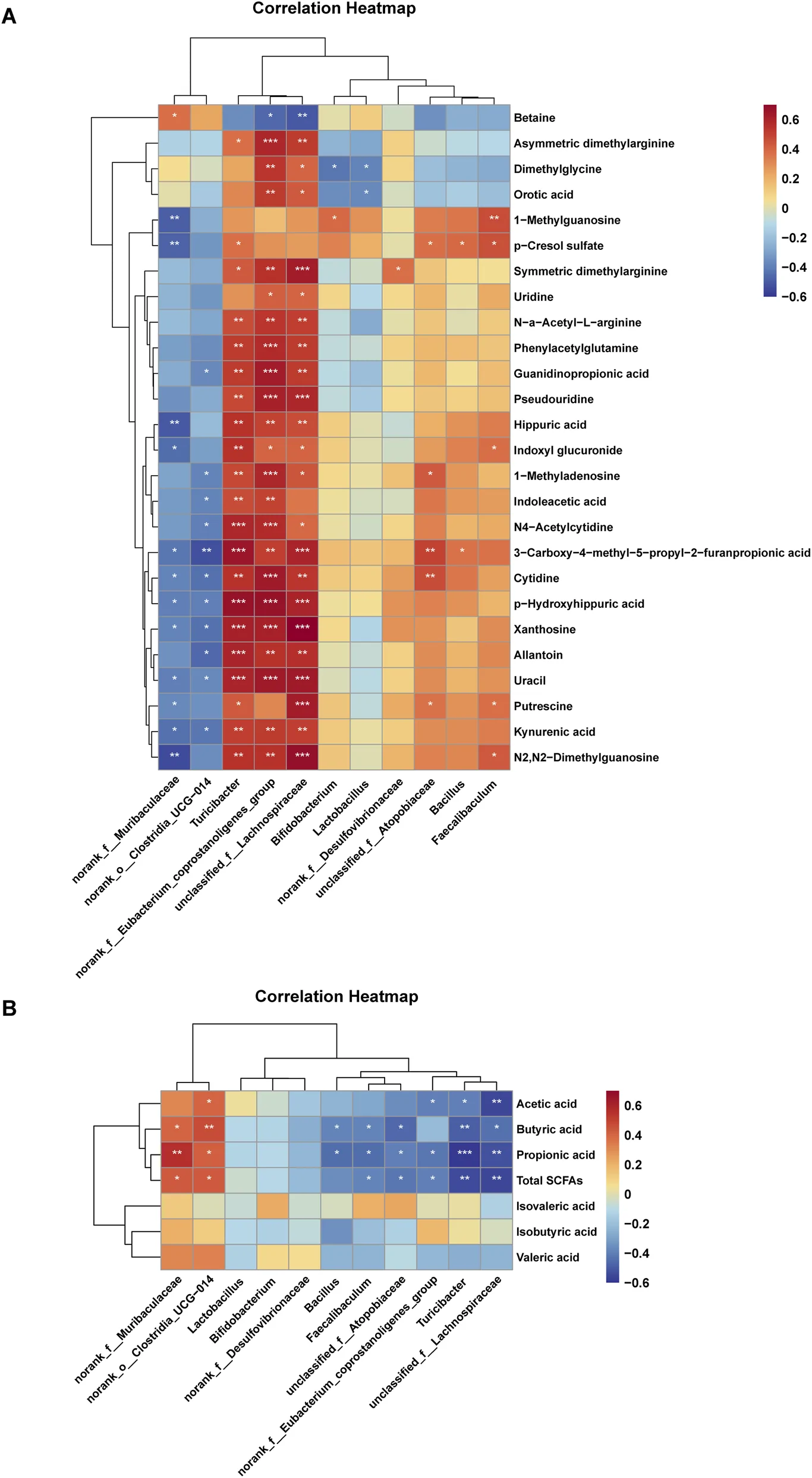
Spearman correlation analysis. **(A)** Correlation heatmap between characteristic genera and plasma kidney disease-related metabolites. **(B)** Correlation heatmap between characteristic genera and fecal SCFAs. (n = 6). *p < 0.05, **p < 0.01, ***p < 0.001.

Investigation of the correlation between plasma metabolic imbalances (focusing on kidney disease-related metabolites), SCFA metabolism aberrations, and intestinal microbial perturbations in CKD mice was conducted using Spearman correlation analysis (Figures 10A,B). The results showed a strong association between gut microbiota and metabolites. Among them, *Turicibacter*, *unclassified_f__ Lachnospiraceae* and *norank_f__Eubacterium_coprostanoligenes_group* exhibited significant positive correlations with uremic toxins such as N2,N2-dimethylguanosine, kynurenic acid, uracil, allantoin, xanthosine, p-hydroxyhippuric acid, cytidine, CMPF, N4-acetylcytidine, pseudouridine and guanidinopropionic acid. Conversely, these genera showed significant negative correlations with beneficial metabolites such as betaine, acetic acid, butyric acid, propionic acid, and total SCFAs. *norank_f__Muribaculaceae* and *norank_o__Clostridia_UCG-014* showed inverse correlations with uremic toxins, whereas their levels were positively associated with health-promoting metabolites including betaine and SCFAs. Furthermore, the genus *Lactobacillus* showed a marked inverse association with dimethylglycine and orotic acid. Overall, these correlative findings suggest that gut microbes are significantly associated with CKD onset and progression, and that intervention with AOP, NKT, or their combination influences these microbial and metabolic associations.

To further assess the association between microbial and metabolic alterations and renal dysfunction, correlations of renal function parameters (BUN and CRE) with kidney disease-related metabolites, characteristic genera, and fecal SCFAs were analyzed (Supplementary Figure S3). Most kidney disease-related plasma metabolites were significantly positively correlated with BUN and CRE levels. Several genera enriched in CKD mice, including *Turicibacter*, *unclassified_f__* *Lachnospiraceae* and *norank_f__Eubacterium_coprostanoligenes_group*, also showed significant positive correlations with renal function parameters. In contrast, individual SCFAs (acetic acid, propionic acid, and butyric acid) as well as total SCFAs were significantly negatively correlated with BUN and CRE. Collectively, these findings help identify specific genera and metabolites closely associated with renal function parameters, further supporting the involvement of the gut-kidney axis in CKD.

## 4 Discussion

Adenine-induced mouse models present the main pathological features of clinical CKD, including renal fibrosis, renal dysfunction, inflammatory responses, gut microbiota dysbiosis, and metabolic disorders (Wei et al., 2024; Wang et al., 2022). This study systematically explored the combined effects of high-molecular-weight polysaccharide (AOP) and low-molecular-weight sesquiterpenoid (NKT), two major metabolites of *A. oxyphylla* fructus. Taking the gut-kidney axis as the core of this research, we evaluated the protective effects of the two metabolites alone or in combination on CKD. We found that the combination of AOP and NKT exerted stronger protective effects than either agent alone by reducing renal and colonic pathological damage in CKD mice, alleviating systemic inflammation, inhibiting the accumulation of potentially harmful microbiota and uremic toxins, increasing SCFA levels, and enhancing colonic barrier function.

After intervention with AOP, NKT, or their combination, inflammation and pathological damage in both the kidneys and colons of CKD mice were concurrently alleviated, which is consistent with the key feature of the interactive regulation of the gut-kidney axis as reported in previous studies (Shen et al., 2023). Additionally, we observed that AOP inhibited the expression of inflammatory factors in colonic tissue and regulated total SCFAs more significantly compared to NKT alone. This suggests that polysaccharide metabolites may offer distinct advantages in regulating intestinal inflammation and the local immune response. Existing literature supports this, highlighting the intestinal-targeting effects of polysaccharides. For example, Yu et al. demonstrated that ICPC-a (Imperata Cylindrica polysaccharide) as a prebiotic markedly modulated the intestinal environment, significantly reducing colonic inflammation and improving colon tissue damage to alleviate hyperuricemia (Yu et al., 2024). According to Chen et al., WHBP (White hyacinth bean polysaccharides) displayed antidiabetic activity by alleviating oxidative stress within the intestine and normalizing epithelial barrier function (Chen et al., 2023). Moreover, multiple studies have confirmed that polysaccharides can alter the intestinal environment via modulation of the gut microbial community structure, influencing the intestinal mucosal immune microenvironment and affecting the activity of critical metabolic enzymes in the gut, and ultimately influencing the absorption and metabolism of low-molecular-weight substances (Fu et al., 2020; Yang et al., 2022; Zhang A. N. et al., 2025; Lian et al., 2025). The more prominent protective effect of the combined intervention over NKT alone in this study may be attributed, at least in part, to AOP-mediated improvement of the intestinal microenvironment, which may create favorable conditions for the renoprotective effect of NKT.

Metabolomics analyses showed significant changes in CKD mouse plasma metabolic profiles. Decreased renal filtration led to the accumulation of large amounts of endogenous metabolites in the plasma. In particular, the accumulation of uremic toxins has become an important factor in damaging renal function and inducing complications (Ravid et al., 2021). Compared with AOP or NKT administration alone, the combined AOP-NKT administration had a broader impact on metabolic pathways, including those related to nucleotide metabolism (pyrimidine metabolism, purine metabolism), amino acid metabolism (phenylalanine metabolism, phenylalanine-tyrosine and tryptophan biosynthesis, and glycine-serine and threonine metabolism), and those pathways implicated in oxidative stress (ascorbate and aldarate metabolism and one-carbon pool by folate). The above pathways are highly overlapping with those previously reported to be associated with metabolic disorders in CKD (Chen et al., 2017; Cai et al., 2018). The combined intervention group more comprehensively corrected the metabolic disorder network in CKD mice.

In addition, we observed that among the identified kidney disease-related metabolites, except for betaine, all showed an accumulation trend in the MOD group and this accumulation pattern diminished following administration of AOP, NKT, or their combination, especially in the AOP-NKT group. A reduction in betaine concentrations was observed during CKD progression, consistent with previous reports. Betaine is considered an important marker for identifying different CKD stages and exhibits strong links to renal inflammation, oxidative stress and gut microbiota dysbiosis (Alvarenga et al., 2022). In addition, nine kidney disease-related metabolites, including p-cresol sulfate, indoleacetic acid, hippuric acid, and CMPF, were exclusively identified as differential metabolites in the AOP-NKT group compared to NKT group. Among them, p-cresol sulfate, a gut microbiota-derived metabolite of tyrosine and phenylalanine, promotes renal injury by augmenting NADPH oxidase catalytic activity, enhancing reactive oxygen species (ROS) production, and upregulating inflammatory genes associated with renal fibrosis (Watanabe et al., 2013). Indoleacetic acid, an indole-type uremic toxin, can activate downstream signaling pathways mediated by AHR (including MAPK, p38 MAPK/NF-κB), triggering oxidative stress, inflammatory responses and vascular endothelial injury, which are linked to endothelial dysfunction and cardiovascular risk in CKD (Dou et al., 2015). Hippuric acid induces oxidative stress by interfering with the NRF2-KEAP1-CUL3 interaction, thereby activating the ROS-mediated TGF-β/SMAD signaling pathway and ultimately promoting renal fibrosis (Sun et al., 2020). CMPF leads to renal injury by disrupting iron metabolism, inhibiting GPX4 activity, inducing lipid peroxidation-mediated ferroptosis, and triggering apoptosis (Park et al., 2023). In summary, we hypothesize that the renoprotective effect of the AOP-NKT combination is closely associated with the improvement of key uremic toxins.

The key differential metabolites identified in this study, such as p-cresol sulfate and indoleacetic acid, belong to the class of gut-derived uremic toxins, and their production largely arises from the metabolic activities of the intestinal microbial community. In recent years, intervention strategies that target gut microbiota regulation have become a major focus in the management of kidney-related diseases (Bloom et al., 2025; He et al., 2025). Our findings demonstrated that substantial alterations in intestinal microbiome composition were observed in CKD mouse models. At the genus level, *Turicibacter*, *norank_f__Eubacterium_coprostanoligenes_group,* and *unclassified_f__* *Lachnospiraceae* were identified as signature genera in the CKD mice, all exhibiting significantly higher relative abundances compared to the CON group. Notably, *Turicibacter* is closely associated with CKD progression and has been identified in previous studies as a characteristic genus of CKD (Hartjes et al., 2025). In another study on hyperuricaemia, this genus was significantly correlated with uric acid levels (Xu et al., 2025). Meanwhile, the elevated levels of colonic inflammation, along with the upregulation of *Turicibacter* abundance observed in the present study, correspond to previous findings of its enrichment in colitis models (Rahman et al., 2023). *norank_f__Eubacterium_coprostanoligenes_group* was observed to accumulate significantly in the rheumatoid arthritis (RA) study and research on colitis has confirmed its positive correlation with pro-inflammatory cytokines including IL-1β, IL-6, and IFNγ, suggesting that this genus potentially exerts pivotal effects on inflammation (He et al., 2023; Zhang S. S. et al., 2025). *unclassified_f__* *Lachnospiraceae* is thought to have a dual role: it plays a beneficial role in regulating SCFA levels, while its abnormal enrichment is associated with inflammation and metabolic disorders in various pathological models (Fang et al., 2022; Li et al., 2022; Fan et al., 2025). After drug intervention, the abundance of the above three genera showed a decreasing trend or was significantly reduced. We also observed the enrichment of beneficial genera *Clostridium_sensu_stricto_1* and *Lactobacillus* among the pharmacologically treated mice (Wei et al., 2023; Miao et al., 2024b). Drug intervention can effectively downregulate the abundance of potential pathogenic bacteria, enriched beneficial bacteria, and attenuated CKD advancement via modulating the gut microbiota structure.

In this work, the MOD group exhibited a marked decline in acetic, propionic, and butyric acids, as well as total SCFA levels. These alterations were accompanied by marked exacerbation of intestinal inflammation coupled with compromised intestinal barrier function, consistent with pathological features reported in previous CKD studies (Xia et al., 2024; Chen et al., 2022). Accumulating evidence indicates that SCFAs mediate anti-inflammatory effects via activating of FFAR2 and FFAR3, thereby inhibiting downstream inflammation-related signaling pathways (Huang et al., 2021). SCFAs have also been shown to alleviate ethanol-related intestinal hyperpermeability and metabolic strain by activating AMPK (Elamin et al., 2013). Furthermore, SCFAs play a central role in colonic oxygen metabolism by inducing “physiological hypoxia” in the intestinal epithelium, which stabilizes HIF and enhances intestinal barrier integrity, with butyric acid exhibiting the highest potency (Kelly et al., 2015). In our study, drug intervention significantly attenuated the reduction in SCFA levels, especially in the AOP-NKT group, which may contribute to improved intestinal barrier function and subsequent amelioration of CKD-associated “leaky gut”.

We acknowledge several limitations of this study. Although integrated multi-omics analyses revealed an association network linking gut microbiota, SCFAs, plasma metabolomic profiles, and renal function parameters, the observed relationships were mainly correlative in nature. Future functional validation experiments, including antibiotic intervention, fecal microbiota transplantation, or targeted modulation of SCFAs, will help to establish causality and determine whether modulation of the gut-kidney axis mediates the renoprotective effects of AOP and NKT in CKD.

## 5 Conclusion

The present investigation assessed the protective effects of two bioactive metabolites, AOP and NKT, from the traditional Chinese medicine *A. oxyphylla* fructus, administered alone and in combination, in adenine-induced CKD mice. Collectively, these results suggested that the AOP-NKT combined intervention has a significant protective effect advantage compared to single-metabolite administration. The combined administration of AOP and NKT attenuated renal inflammation and fibrosis progression by correcting metabolic disorders, improving the intestinal microenvironment and reducing uremic toxin accumulation. This outcome may be attributed to rebalancing the gut microbiota, thereby maintaining the homeostasis of SCFAs and attenuating barrier dysfunction. These findings indicate that the major active metabolites AOP and NKT in *A. oxyphylla* fructus may exert a combined renoprotective effect on CKD through the gut-kidney axis. In conclusion, this study not only provides experimental support for the renoprotective application of *A. oxyphylla* fructus in CKD but also demonstrates a combined interaction between its metabolites AOP and NKT, offering insight into the multi-metabolite combined potential of traditional Chinese medicine.

## Data Availability

The original contributions presented in the study are publicly available. The metabolomics data can be found here: https://doi.org/10.6084/m9.figshare.33365325. The microbial diversity sequencing data can be found here: https://www.ncbi.nlm.nih.gov/bioproject/PRJNA1498576.
