# Supplementary material for: Gut microbiota and metabolomics reveal the combined effects of Alpinia oxyphylla fructus polysaccharide and nootkatone on chronic kidney disease via the “gut-kidney” axis
Source: Front Pharmacol. 2026 Sep 8;17:1904155. doi: 10.3389/fphar.2026.1904155 (PMC13593784; doi:10.3389/fphar.2026.1904155)
Supplement: Supplementary file 1 [file DataSheet1.pdf]

Supplementary Text S1

Structural and physicochemical characterization of *Alpinia oxyphylla* fructus polysaccharide

The structural and physicochemical characteristics of *Alpinia oxyphylla* fructus polysaccharide (AOP) used in the present study were systematically investigated in our previously published work (Int. J. Biol. Macromol., 2025, 292, 139367; https://doi.org/10.1016/j.ijbiomac.2024.139367).

AOP was a slightly brownish-yellow, homogeneous acidic polysaccharide with a molecular weight of 1151.9 kDa. Its protein, total sugar, and uronic acid contents were 0.63%, 41.02%, and 75.44%, respectively. Monosaccharide composition analysis (mol%) revealed that AOP consisted of mannose (0.71%), rhamnose (2.05%), glucuronic acid (2.41%), galacturonic acid (31.39%), glucose (33.31%), galactose (8.54%), xylose (10.50%), and arabinose (11.10%), with glucose and galacturonic acid as the predominant monosaccharides. FT-IR analysis indicated that AOP was a pyranose-type acidic polysaccharide with β-glycosidic bonds.

Supplementary Text S2

Chromatographic and mass-spectrometric parameters

Chromatographic parameters: 5 μL of sample was separated at 40 °C on an ACQUITY UPLC HSS T3 column (1.8 μm, 2.1 × 100 mm, Waters, USA) at a flow rate of 0.3 mL/min. The mobile phase consisted of acetonitrile (A) and 0.1% formic acid (B). The 20-minute gradient program was as follows: 0–1 min, 5–20% A; 1–3 min, 20–40% A; 3–5 min, 40–60% A; 5–11 min, 60–70% A; 11–14 min, 70–95% A; 14–16 min, 95% A; 16–18 min, 95–5% A; 18–20 min, 5% A.

Mass spectrometry parameters: The electrospray ionization (ESI) source was operated in positive/negative ion mode with the following parameters: Ion Source Gas 1 (GS1), 50 psi; Ion Source Gas 2 (GS2), 50 psi; Curtain Gas (CUR), 30 psi; Temperature (TEM), 550 °C; Ion Spray Voltage Floating (ISVF), 5500/–4500 V; Declustering Potential (DP), ±80 V; Collision Energy (CE), ±35 eV; Mass scan range m/z: 50–1000 (positive) and 100–1000 (negative) Da.

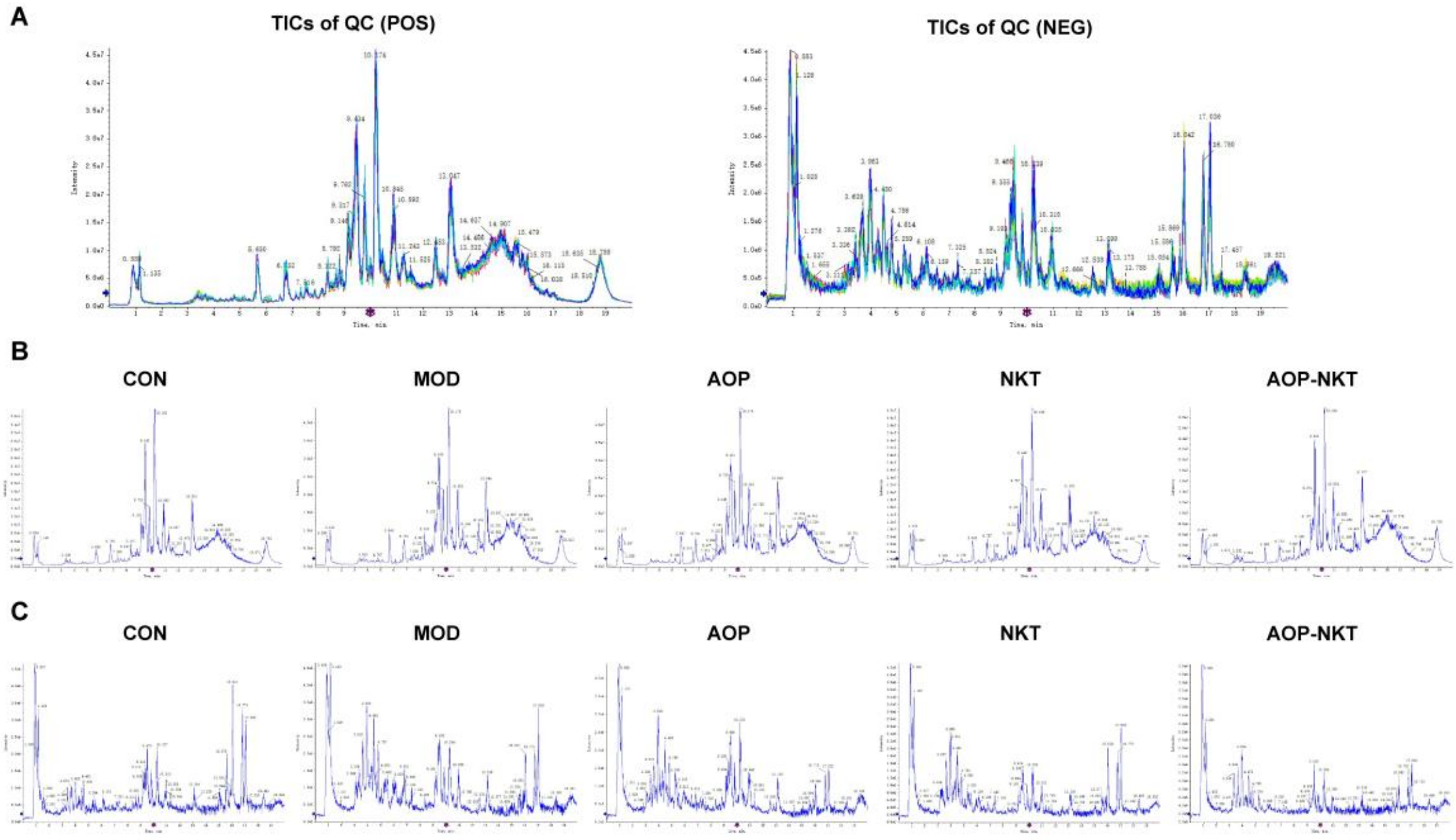

Fig. S1 **(A)** TICs in positive and negative ion modes of QC samples. **(B)** Representative TICs in positive ion mode of plasma samples from each experimental group. **(C)** Representative TICs in negative ion mode of plasma samples from each experimental group.

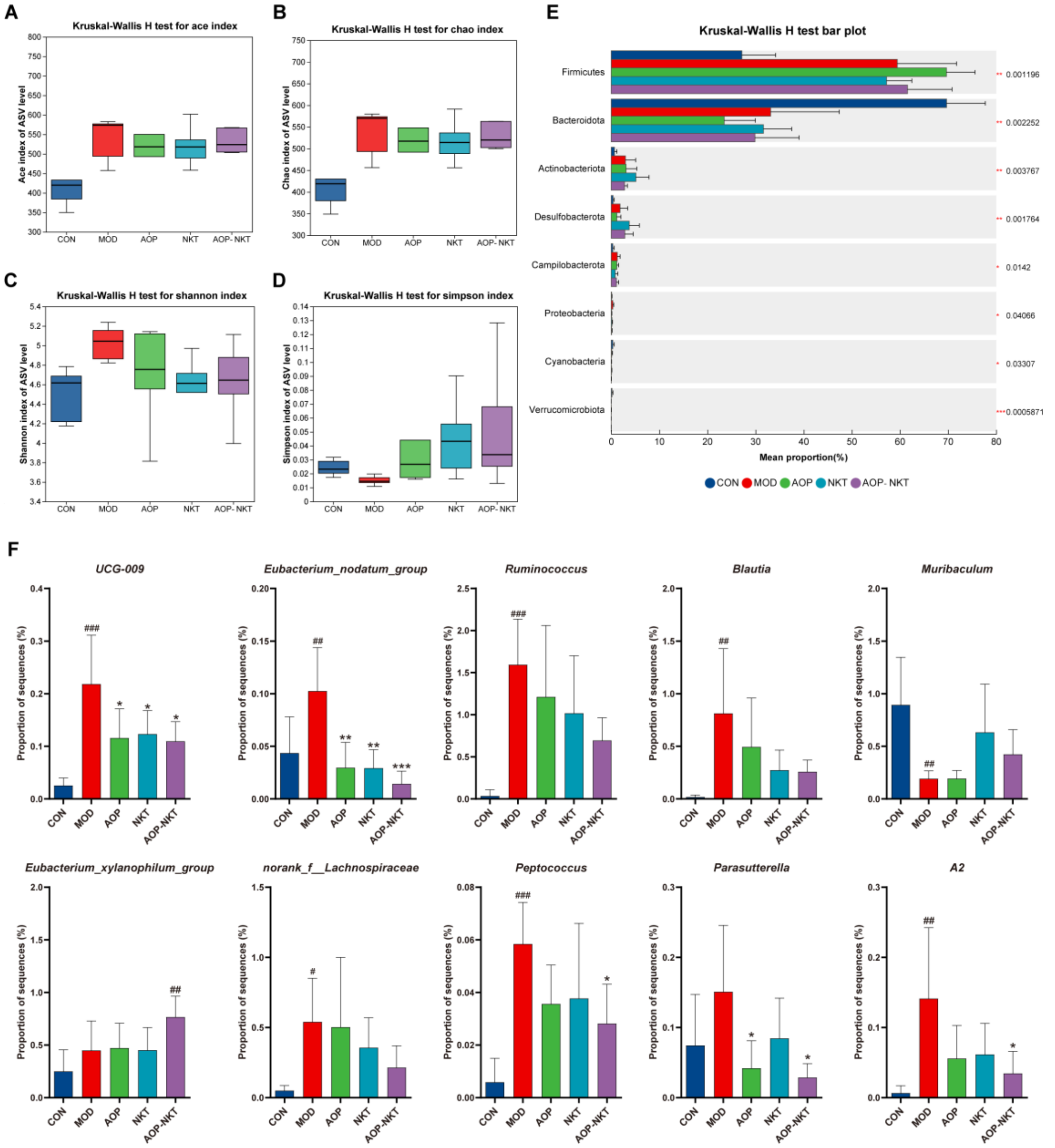

Fig. S2 Alpha diversity analysis and differential taxa. **(A-D)** Alpha diversity analysis includes Ace **(A)**, Chao **(B)**, Shannon **(C)**, and Simpson **(D)** indices. **(E)** Comparison of relative abundance of the gut microbiome at the phylum level. **(F)** Relative abundance of representative differential genera. Data are expressed as mean ± SD (n = 6). # $p < 0.05$, ## $p < 0.01$, ### $p < 0.001$ vs. CON group; * $p < 0.05$, ** $p < 0.01$, *** $p < 0.001$ vs. MOD group.

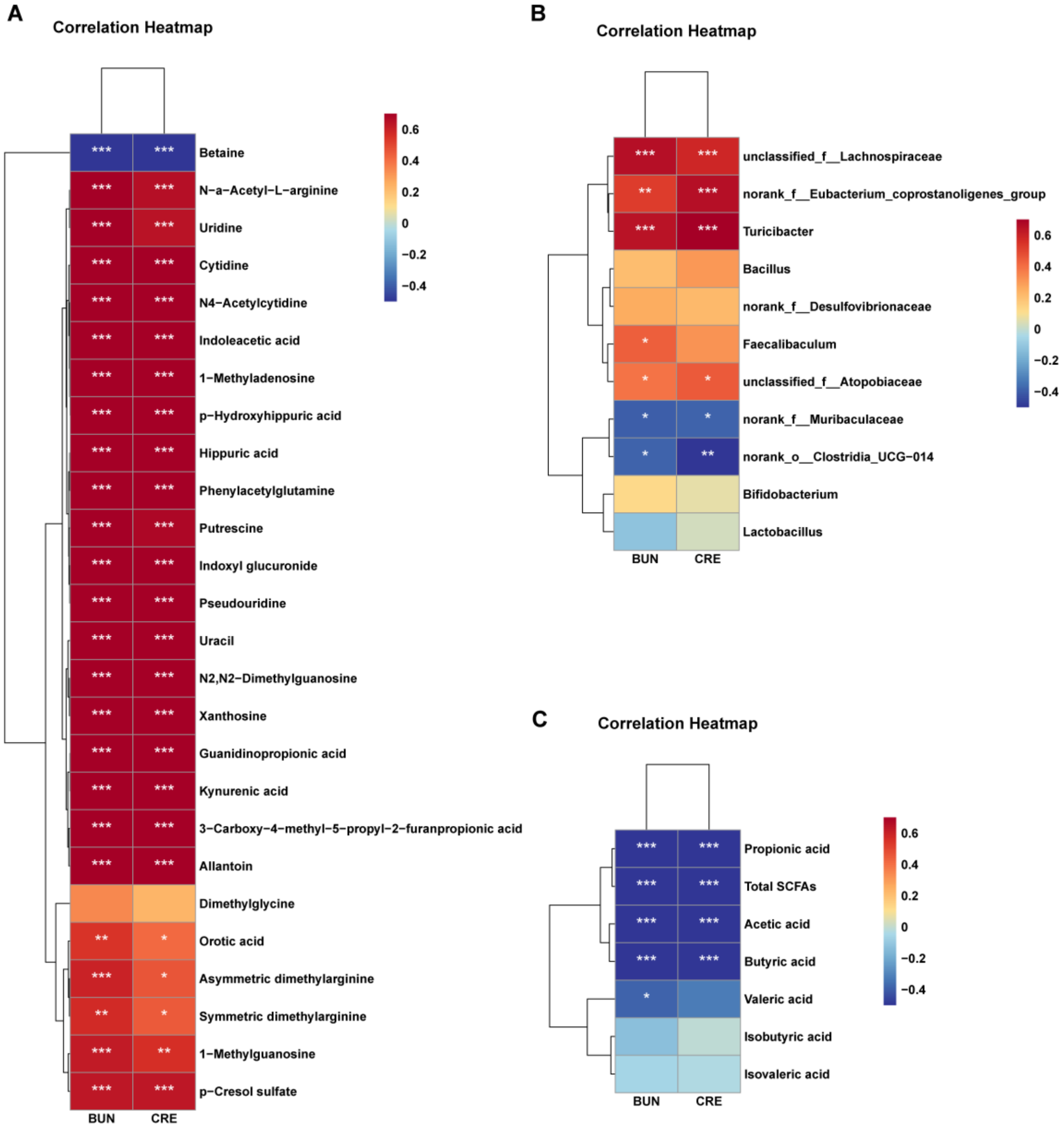

Fig. S3 Spearman correlation analysis. **(A)** Correlation heatmap between renal function parameters and plasma kidney disease-related metabolites. **(B)** Correlation heatmap between renal function parameters and characteristic genera. **(C)** Correlation heatmap between renal function parameters and fecal SCFAs. ($n = 6$). * $p < 0.05$, ** $p < 0.01$, *** $p < 0.001$.

Table S1 Primer sequences for RT-qPCR analysis.

Gene		Primer Sequence(5'-3')
TNF-α	Forward	ACGGCATGGATCTCAAAGAC
	Reverse	AGATAGCAAATCGGCTGACG
IL-1β	Forward	TATGGGCTGGACTGTTTCTA
	Reverse	CATCAGAGGCAAGGAGGAA
IL-6	Forward	TAGTCCTTCCTACCCCAATTTCC
	Reverse	TTGGTCCTTAGCCACTCCTTC
ZO-1	Forward	GATGAGCGGGCTACCTTA
	Reverse	TGGAGACTGCGTGGAATG
occludin	Forward	TTTCCTGCGGTGACTTCT
	Reverse	AATGTATCGTATTTCCCCTGA
claudin-1	Forward	GGTGTCCTACTTTCCTGCTC
	Reverse	TGTTCTACCACAGTTCCGATA
muc2	Forward	ACCTCCAACTGAATCCTCG
	Reverse	TGTGGTTACAGGGGTGCTT
β-actin	Forward	GTGACGTTGACATCCGTAAAGA
	Reverse	GCCGGACTCATCGTACTCC

Table S2 Differential metabolites in plasma after AOP treatment.

NO.	m/z	Ret. Time (min)	HMDB ID	Metabolites	POS/NEG	Formula	CON VS. MOD			AOP VS. MOD		
							p-value	VIP	FC	p-value	VIP	FC
1	88.047	5.67	HMDB0000243	Pyruvic acid	POS	C3H4O3	0.0001	1.4541	2.20999	0.03641	1.5357	1.24994
2	88.1569	5.65	HMDB0001414	Putrescine	POS	C4H12N2	5.3E-05	1.9773	2.37596	0.01696	2.3236	1.31788
3	89.0221	5.6	HMDB0000056	beta-Alanine	POS	C3H7NO2	8.7E-06	1.0706	3.71076	0.00222	1.4188	1.5339
4	118.0649	3.38	HMDB0000043	Betaine	POS	C5H12NO2	7E-06	1.1738	0.62415	9.1E-05	1.6921	0.7536
5	142.1591	5.35	HMDB0240650	Ectoine	POS	C6H10N2O2	3E-05	1.3222	0.69253	4.4E-05	2.1016	0.78753
6	203.0526	0.92	HMDB0060484	Indolepyruvate	POS	C11H9NO3	0.01075	1.6531	0.68863	0.00013	6.7026	0.40787
7	240.094	1.12	HMDB0061112	3-Carboxy-4-methyl-5-propyl-2-furanpropionic acid	POS	C12H16O5	3.1E-05	1.0492	8.44132	0.00462	1.4872	1.83404
8	245.2268	14.93	HMDB0000688	Isovalerylcarnitine	POS	C12H23NO4	5.8E-07	2.3847	3.21978	7.5E-05	3.6341	1.66454
9	276.1272	3.86	HMDB0000279	Saccharopine	POS	C11H20N2O6	0.00023	1.067	4.76815	0.00876	1.4829	1.93103
10	300.201	3.48	HMDB0001852	all-trans-Retinoic acid	POS	C20H28O2	6.7E-05	2.6476	2.85961	0.00437	3.5791	1.48702
11	368.2795	7.09	HMDB0002003	Tetracosanoic acid	POS	C24H48O2	0.00066	1.3988	1.77583	0.00125	2.5682	1.49068
12	379.2824	14.82	HMDB0000277	Sphingosine 1-phosphate	POS	C18H38NO5P	9E-08	2.1523	6.16406	0.00038	2.6591	1.46672
13	396.3098	7.82	HMDB0002356	Hexacosanoic acid	POS	C26H52O2	0.00701	1.3929	2.01529	0.00457	3.0652	2.00619
14	402.3927	10.51	HMDB0001496	7alpha-Hydroxycholesterol	POS	C27H46O2	0.00042	2.0184	3.47125	0.03748	2.5049	1.48638
15	103.0384	1.67	HMDB0000092	Dimethylglycine	NEG	C4H9NO2	2.2E-07	4.0548	1.88714	4.3E-08	6.6845	2.41213
16	111.0079	1.13	HMDB0000630	Cytosine	NEG	C4H5N3O	7.1E-10	4.1004	3.00577	0.00697	3.3472	1.37017
17	111.9443	3.96	HMDB0000300	Uracil	NEG	C4H4N2O2	7.6E-09	2.4341	4.63251	0.00199	1.7205	1.28325
18	117.0537	3.06	HMDB0000532	Acetylglycine	NEG	C4H7NO3	1.8E-09	6.1226	2.34262	1.8E-07	7.1027	1.55331

NO.	m/z	Ret. Time (min)	HMDB ID	Metabolites	POS/NEG	Formula	CON VS. MOD			AOP VS. MOD		
							p-value	VIP	FC	p-value	VIP	FC
19	117.0541	2.78	HMDB0000738	Indole	NEG	C8H7N	2.9E-07	2.2145	2.95742	3.9E-05	2.4818	1.66188
20	119.0327	1.01	HMDB0000167	L-Threonine	NEG	C4H9NO3	6.2E-07	2.0151	2.46711	0.00013	2.3934	1.68839
21	124.9887	0.91	HMDB0000251	Taurine	NEG	C2H7NO3S	4.5E-08	1.9706	8.1101	1.3E-05	2.2243	2.19604
22	129.0172	1.12	HMDB0000267	Pyroglutamic acid	NEG	C5H7NO3	2.5E-09	2.6222	3.31722	7.7E-05	2.7546	1.62974
23	131.0706	4.21	HMDB0013222	Guanidinopropionic acid	NEG	C4H9N3O2	2.8E-08	5.2289	2.79646	0.00141	4.9187	1.45364
24	135.0268	0.93	HMDB0000034	Adenine	NEG	C5H5N5	1.6E-08	2.8567	2.34165	5.1E-06	3.1288	1.4987
25	142.0491	1.75	HMDB0000469	5-Hydroxymethyluracil	NEG	C5H6N2O3	3.7E-09	2.2902	4.16348	3.6E-07	3.4424	5.0096
26	144.064	1.94	HMDB0000482	Caprylic acid	NEG	C8H16O2	1.6E-07	2.588	3.2402	9.9E-06	3.3706	2.22607
27	149.0432	0.98	HMDB0000696	Methionine	NEG	C5H11NO2S	2.2E-09	2.4596	15.8442	0.00054	1.7627	1.34812
28	155.1053	5.28	HMDB0000177	Histidine	NEG	C6H9N3O2	4.6E-11	1.914	5.96557	4.6E-07	2.3356	2.41991
29	156.0651	3.35	HMDB0000226	Orotic acid	NEG	C5H4N2O4	7.8E-08	1.8211	3.46542	6.4E-06	2.5954	3.24273
30	157.0341	0.96	HMDB0000959	Tiglylglycine	NEG	C7H11NO3	4.3E-11	3.253	4.9794	0.00011	2.2998	1.2535
31	157.0474	3.4	HMDB0000459	3-Methylcrotonylglycine	NEG	C7H11NO3	2.6E-09	1.104	4.03587	4.2E-05	1.161	1.72079
32	158.0807	3.47	HMDB0003349	L-Dihydroorotic acid	NEG	C5H6N2O4	1.4E-06	2.8395	2.63979	0.00021	3.3142	1.73983
33	161.0431	1.15	HMDB0000510	Aminoadipic acid	NEG	C6H11NO4	7E-06	1.3075	1.79494	8.5E-07	1.9488	1.82582
34	166.0158	0.98	HMDB0000779	Phenyllactic acid	NEG	C9H10O3	3.6E-08	3.3073	3.60188	4.9E-06	3.8756	1.88985
35	172.9891	4.93	HMDB0000842	Quinaldic acid	NEG	C10H7NO2	4.4E-08	1.6901	3.84046	0.00144	1.5611	1.52981
36	173.0051	1.13	HMDB0000701	Hexanoylglycine	NEG	C8H15NO3	1.3E-11	4.7558	4.2072	9E-05	4.8609	1.6687
37	175.0028	3.59	HMDB0000812	N-Acetyl-L-aspartic acid	NEG	C6H9NO5	4E-08	1.4571	3.25958	0.00014	1.4903	1.58723
38	175.0217	4.31	HMDB0000904	Citrulline	NEG	C6H13N3O3	1.2E-08	1.3519	5.58348	2.6E-06	1.5035	1.92999

NO.	m/z	Ret. Time (min)	HMDB ID	Metabolites	POS/NEG	Formula	CON VS. MOD			AOP VS. MOD		
							p-value	VIP	FC	p-value	VIP	FC
39	181.0481	3.28	HMDB0000158	L-Tyrosine	NEG	C9H11NO3	2.7E-07	5.2049	5.33656	0.0002	5.6657	1.98176
40	182.0513	3.28	HMDB0000107	Galactitol	NEG	C6H14O6	4.7E-07	1.6615	5.4489	0.00017	1.8297	2.01493
41	184.1018	4.55	HMDB0001565	Phosphorylcholine	NEG	C5H15NO4P	4E-09	1.3069	3.10352	0.02936	1.0726	1.48649
42	185.0788	4.85	HMDB0000272	Phosphoserine	NEG	C3H8NO6P	4E-10	1.3456	3.80656	6.4E-06	1.5893	1.99651
43	186.0731	1.52	HMDB0000807	3-Phosphoglyceric acid	NEG	C3H7O7P	0.00058	1.0495	8.50059	0.00085	1.5437	6.69174
44	190.0032	4.46	HMDB0001370	Diaminopimelic acid	NEG	C7H14N2O4	1E-06	1.1386	4.76352	0.00039	1.1636	1.69282
45	191.0167	5.56	HMDB0011745	N-Acetyl-L-methionine	NEG	C7H13NO3S	2.7E-10	2.3417	2.36633	6.4E-05	2.2861	1.38362
46	192.0645	4.25	HMDB0000193	Isocitric acid	NEG	C6H8O7	8.1E-08	7.3577	5.7239	0.00736	4.9439	1.29606
47	193.0657	4.25	HMDB0000821	Phenylacetylglycine	NEG	C10H11NO3	0.00019	2.7346	6.51907	0.03934	2.1614	1.55013
48	194.0428	3.21	HMDB0000127	D-Glucuronic acid	NEG	C6H10O7	9.3E-09	1.4575	11.5523	0.00018	1.631	2.41011
49	195.0486	0.91	HMDB0013678	p-Hydroxyhippuric acid	NEG	C9H9NO4	1E-07	3.6897	5.84948	0.00958	2.3476	1.27471
50	197.0791	4.8	HMDB0032055	N-Acetylhistidine	NEG	C8H11N3O3	1.1E-05	2.2175	2.3763	0.03009	2.1744	1.5942
51	202.0675	2.46	HMDB0003334	Symmetric dimethylarginine	NEG	C8H18N4O2	2.9E-09	1.2151	4.36248	1.2E-06	1.6597	3.1375
52	202.1054	3.31	HMDB0001539	Asymmetric dimethylarginine	NEG	C8H18N4O2	7.1E-08	1.005	4.42629	2.7E-06	1.4117	3.44999
53	204.0646	4.81	HMDB0000929	L-Tryptophan	NEG	C11H12N2O2	3.4E-08	9.399	7.72363	0.03632	5.428	1.27958
54	204.9799	3.64	HMDB0011621	Cinnamoylglycine	NEG	C11H11NO3	1.3E-05	1.2131	3.2584	0.00674	1.1166	1.52438
55	210.0408	1.12	HMDB0000663	Glucaric acid	NEG	C6H10O8	4.9E-05	1.1774	6.39003	5.8E-05	1.7182	6.44045
56	215.0336	5.93	HMDB0001885	3-Chlorotyrosine	NEG	C9H10ClNO3	3.5E-08	1.295	4.83498	4.1E-05	1.6589	2.78967
57	216.0835	2.9	HMDB0004620	N-a-Acetyl-L-arginine	NEG	C8H16N4O3	8.8E-08	1.0069	3.05661	3.3E-05	1.2661	2.05988

NO.	m/z	Ret. Time (min)	HMDB ID	Metabolites	POS/NEG	Formula	CON VS. MOD			AOP VS. MOD		
							p-value	VIP	FC	p-value	VIP	FC
58	216.0954	1.13	HMDB0000888	Undecanedioic acid	NEG	C11H20O4	0.00022	1.0216	8.67996	0.00442	1.1226	2.36755
59	220.0575	4.77	HMDB0000472	5-Hydroxy-L-tryptophan	NEG	C11H12N2O3	9.5E-07	1.803	8.00004	0.01526	1.351	1.48772
60	221.0742	5.29	HMDB0000215	N-Acetyl-D-glucosamine	NEG	C8H15NO6	5.4E-08	1.0105	2.70659	3.4E-05	1.2155	1.80493
61	223.9976	3.89	HMDB0000732	Hydroxykynurenine	NEG	C10H12N2O4	3.3E-07	3.1913	8.39051	0.00121	2.8311	1.59688
62	227.1246	6.17	HMDB0000014	Deoxycytidine	NEG	C9H13N3O4	1.3E-05	2.0732	1.94613	0.04228	1.5974	1.25008
63	228.0836	3.5	HMDB0006695	Prolylhydroxyproline	NEG	C10H16N2O4	0.00122	1.2061	1.63125	0.01209	1.5243	1.50389
64	243.059	1.01	HMDB0000089	Cytidine	NEG	C9H13N3O5	2.5E-08	3.4302	3.97544	7.5E-05	3.1999	1.46747
65	244.1157	4.64	HMDB0000767	Pseudouridine	NEG	C9H12N2O6	1.9E-09	1.288	3.03213	0.00151	1.2361	1.51765
66	251.0919	5.49	HMDB0000101	Deoxyadenosine	NEG	C10H13N5O3	1.1E-05	1.8817	17.5767	0.00142	1.9091	2.10876
67	257.0995	3.15	HMDB0000086	Glycerophosphocholine	NEG	C8H20NO6P	1.9E-06	1.6873	2.61309	0.00013	1.9396	1.64406
68	260.0211	1.13	HMDB0001401	Glucose 6-phosphate	NEG	C6H13O9P	1.1E-08	1.4982	5.28314	0.00364	1.0082	1.27426
69	262.0044	3.25	HMDB0011719	Homovanillic acid sulfate	NEG	C9H10O7S	1.4E-09	1.3954	11.7552	0.00191	1.1052	1.4921
70	264.0101	1.96	HMDB0006344	Phenylacetylglutamine	NEG	C13H16N2O4	1.1E-06	1.3461	2.959	0.00013	1.6114	1.85392
71	276.087	4.64	HMDB0001316	6-Phosphogluconic acid	NEG	C6H13O10P	4.4E-10	1.0558	8.07816	9.5E-05	1.1269	2.03182
72	281.0518	1.06	HMDB0003331	1-Methyladenosine	NEG	C11H15N5O4	6.6E-06	1.193	3.09737	0.0013	1.2906	1.6644
73	283.0793	4.34	HMDB0000133	Guanosine	NEG	C10H13N5O5	5.9E-08	8.6606	5.23434	1.4E-05	9.6002	1.91019
74	284.0215	3.19	HMDB0000299	Xanthosine	NEG	C10H12N4O6	7.4E-06	1.4206	25.0618	0.00276	1.435	2.13335
75	345.0314	3.27	HMDB0001314	Cyclic GMP	NEG	C10H12N5O7P	3.9E-09	1.4513	9.28436	8.1E-08	1.9183	3.61285

Table S3 Differential metabolites in plasma after NKT treatment.

NO.	m/z	Ret. Time (min)	HMDB ID	Metabolites	POS/NEG	Formula	CON VS. MOD			NKT VS. MOD		
							p-value	VIP	FC	p-value	VIP	FC
1	88.047	5.67	HMDB0000243	Pyruvic acid	POS	C3H4O3	0.0001	1.4541	2.20999	0.01813	1.2403	1.2463
2	88.1569	5.65	HMDB0001414	Putrescine	POS	C4H12N2	5.3E-05	1.9773	2.37596	0.00839	1.9278	1.33629
3	89.0221	5.6	HMDB0000056	beta-Alanine	POS	C3H7NO2	8.7E-06	1.0706	3.71076	0.00877	1.0168	1.46536
4	118.0649	3.38	HMDB0000043	Betaine	POS	C5H12NO2	7E-06	1.1738	0.62415	4.5E-07	1.6666	0.704
5	142.1591	5.35	HMDB0240650	Ectoine	POS	C6H10N2O2	3E-05	1.3222	0.69253	4.2E-06	1.89	0.76006
6	194.0818	4.24	HMDB0002545	Galacturonic acid	POS	C6H10O7	1.2E-05	2.1224	11.8496	0.00567	2.1655	1.84076
7	203.0526	0.92	HMDB0060484	Indolepyruvate	POS	C11H9NO3	0.01075	1.6531	0.68863	0.02772	2.7615	0.64043
8	245.2268	14.93	HMDB0000688	Isovalerylcarnitine	POS	C12H23NO4	5.8E-07	2.3847	3.21978	0.00042	2.9231	1.83
9	285.2411	13.4	HMDB0013324	2-Octenoylcarnitine	POS	C15H27NO4	4.8E-05	1.3373	3.8453	0.0381	1.1338	1.39078
10	300.201	3.48	HMDB0001852	all-trans-Retinoic acid	POS	C20H28O2	6.7E-05	2.6476	2.85961	0.00858	2.609	1.42905
11	374.3621	8.71	HMDB0000308	3b-Hydroxy-5-cholenoic acid	POS	C24H38O3	0.0002	3.3984	3.40203	0.01891	3.5862	1.5446
12	379.2824	14.82	HMDB0000277	Sphingosine 1-phosphate	POS	C18H38NO5P	9E-08	2.1523	6.16406	0.00071	2.3466	1.78573
13	402.3927	10.51	HMDB0001496	7alpha-Hydroxycholesterol	POS	C27H46O2	0.00042	2.0184	3.47125	0.00639	2.6452	1.94958
14	730.539	14.71	HMDB0001348	SM(d18: 1/18: 0)	POS	C41H83N2O6P	0.00112	1.3496	1.78329	0.02074	1.6236	1.497
15	103.0384	1.67	HMDB0000092	Dimethylglycine	NEG	C4H9NO2	2.2E-07	4.0548	1.88714	6.9E-05	5.9282	2.01546
16	103.9184	19.54	HMDB0000691	Malonic acid	NEG	C3H4O4	0.00275	1.3412	1.28056	0.0004	1.8134	1.20247
17	111.0079	1.13	HMDB0000630	Cytosine	NEG	C4H5N3O	7.1E-10	4.1004	3.00577	0.00056	4.115	1.55951
18	111.9443	3.96	HMDB0000300	Uracil	NEG	C4H4N2O2	7.6E-09	2.4341	4.63251	0.00099	1.8764	1.36426
19	117.0537	3.06	HMDB0000532	Acetylglycine	NEG	C4H7NO3	1.8E-09	6.1226	2.34262	4.1E-05	7.4808	1.68539

NO.	m/z	Ret. Time (min)	HMDB ID	Metabolites	POS/NEG	Formula	CON VS. MOD			NKT VS. MOD		
							p-value	VIP	FC	p-value	VIP	FC
20	117.0541	2.78	HMDB0000738	Indole	NEG	C8H7N	2.9E-07	2.2145	2.95742	0.00061	2.3806	1.6313
21	119.0327	1.01	HMDB0000167	L-Threonine	NEG	C4H9NO3	6.2E-07	2.0151	2.46711	0.00152	2.0382	1.47117
22	124.9887	0.91	HMDB0000251	Taurine	NEG	C2H7NO3S	4.5E-08	1.9706	8.1101	0.00012	1.8635	1.64579
23	129.0172	1.12	HMDB0000267	Pyroglutamic acid	NEG	C5H7NO3	2.5E-09	2.6222	3.31722	5.7E-06	3.0971	1.8363
24	131.0706	4.21	HMDB0013222	Guanidinopropionic acid	NEG	C4H9N3O2	2.8E-08	5.2289	2.79646	3.4E-06	6.363	1.77565
25	135.0268	0.93	HMDB0000034	Adenine	NEG	C5H5N5	1.6E-08	2.8567	2.34165	0.00014	2.4751	1.27997
26	142.0491	1.75	HMDB0000469	5-Hydroxymethyluracil	NEG	C5H6N2O3	3.7E-09	2.2902	4.16348	0.00089	2.7963	2.54996
27	144.064	1.94	HMDB0000482	Caprylic acid	NEG	C8H16O2	1.6E-07	2.588	3.2402	0.00012	3.1103	1.92306
28	149.0432	0.98	HMDB0000696	Methionine	NEG	C5H11NO2S	2.2E-09	2.4596	15.8442	0.0005	1.9231	1.4426
29	155.1053	5.28	HMDB0000177	Histidine	NEG	C6H9N3O2	4.6E-11	1.914	5.96557	9.9E-05	1.9236	1.76007
30	156.0651	3.35	HMDB0000226	Orotic acid	NEG	C5H4N2O4	7.8E-08	1.8211	3.46542	0.00046	2.3212	2.42076
31	157.0341	0.96	HMDB0000959	Tiglylglycine	NEG	C7H11NO3	4.3E-11	3.253	4.9794	1.4E-05	2.884	1.44267
32	157.0474	3.4	HMDB0000459	3-Methylcrotonylglycine	NEG	C7H11NO3	2.6E-09	1.104	4.03587	8.3E-06	1.3935	2.3047
33	158.0601	4.78	HMDB0000462	Allantoin	NEG	C4H6N4O3	4.4E-07	1.2829	11.0202	0.00044	1.1962	1.69929
34	158.0807	3.47	HMDB0003349	L-Dihydroorotic acid	NEG	C5H6N2O4	1.4E-06	2.8395	2.63979	6.5E-05	3.5061	1.80241
35	161.0431	1.15	HMDB0000510	Aminoadipic acid	NEG	C6H11NO4	7E-06	1.3075	1.79494	2.3E-06	1.9699	1.86151
36	162.0037	9.21	HMDB0000355	3-Hydroxymethylglutaric acid	NEG	C6H10O5	0.00068	1.2432	11.4778	0.01126	1.3961	2.53679
37	164.0701	4.6	HMDB0000205	Phenylpyruvic acid	NEG	C9H8O3	1.3E-09	1.0645	5.56065	8.2E-06	1.0129	1.55068
38	165.0377	0.93	HMDB0002005	Methionine sulfoxide	NEG	C5H11NO3S	2.5E-07	1.7485	5.4063	0.00387	1.2948	1.36433
39	165.0535	4.6	HMDB0000897	7-Methylguanine	NEG	C6H7N5O	3.5E-06	2.8093	7.66639	0.00383	2.5193	1.68147

NO.	m/z	Ret. Time (min)	HMDB ID	Metabolites	POS/NEG	Formula	CON VS. MOD			NKT VS. MOD		
							p-value	VIP	FC	p-value	VIP	FC
40	166.0158	0.98	HMDB0000779	Phenyllactic acid	NEG	C9H10O3	3.6E-08	3.3073	3.60188	1.4E-05	3.6429	1.7386
41	172.9891	4.93	HMDB0000842	Quinaldic acid	NEG	C10H7NO2	4.4E-08	1.6901	3.84046	2.3E-05	1.663	1.56066
42	173.0051	1.13	HMDB0000701	Hexanoylglycine	NEG	C8H15NO3	1.3E-11	4.7558	4.2072	2.4E-05	5.277	1.85336
43	174.0081	1.12	HMDB0060015	Phenol sulphate	NEG	C6H6O4S	3.5E-08	1.0216	3.05258	0.00065	1.096	1.66868
44	174.0853	0.92	HMDB0000893	Suberic acid	NEG	C8H14O4	1.1E-07	1.1686	4.62007	0.00339	1.0609	1.60536
45	175.0028	3.59	HMDB0000812	N-Acetyl-L-aspartic acid	NEG	C6H9NO5	4E-08	1.4571	3.25958	1.7E-05	1.5439	1.58848
46	175.0217	4.31	HMDB0000904	Citrulline	NEG	C6H13N3O3	1.2E-08	1.3519	5.58348	6.4E-05	1.2786	1.53572
47	181.0481	3.28	HMDB0000158	L-Tyrosine	NEG	C9H11NO3	2.7E-07	5.2049	5.33656	2.7E-05	6.4275	2.42548
48	182.0513	3.28	HMDB0000107	Galactitol	NEG	C6H14O6	4.7E-07	1.6615	5.4489	4.5E-05	2.0406	2.45526
49	184.1018	4.55	HMDB0001565	Phosphorylcholine	NEG	C5H15NO4P	4E-09	1.3069	3.10352	0.00023	1.5794	2.02165
50	185.0788	4.85	HMDB0000272	Phosphoserine	NEG	C3H8NO6P	4E-10	1.3456	3.80656	1.1E-05	1.3973	1.63853
51	186.0731	1.52	HMDB0000807	3-Phosphoglyceric acid	NEG	C3H7O7P	0.00058	1.0495	8.50059	0.02825	1.1219	2.55206
52	190.0032	4.46	HMDB0001370	Diaminopimelic acid	NEG	C7H14N2O4	1E-06	1.1386	4.76352	0.00231	1.0006	1.50789
53	191.0167	5.56	HMDB0011745	N-Acetyl-L-methionine	NEG	C7H13NO3S	2.7E-10	2.3417	2.36633	3.4E-06	2.9618	1.80031
54	192.0645	4.25	HMDB0000193	Isocitric acid	NEG	C6H8O7	8.1E-08	7.3577	5.7239	1.8E-05	7.8979	1.84738
55	193.0657	4.25	HMDB0000821	Phenylacetylglycine	NEG	C10H11NO3	0.00019	2.7346	6.51907	0.00485	2.9312	2.13946
56	194.0428	3.21	HMDB0000127	D-Glucuronic acid	NEG	C6H10O7	9.3E-09	1.4575	11.5523	3.1E-07	1.8173	2.99892
57	195.0486	0.91	HMDB0013678	p-Hydroxyhippuric acid	NEG	C9H9NO4	1E-07	3.6897	5.84948	0.00575	2.4996	1.2936
58	197.0791	4.8	HMDB0032055	N-Acetylhistidine	NEG	C8H11N3O3	1.1E-05	2.2175	2.3763	0.0004	3.0039	2.08233
59	202.0675	2.46	HMDB0003334	Symmetric dimethylarginine	NEG	C8H18N4O2	2.9E-09	1.2151	4.36248	0.00071	1.3452	2.02446

NO.	m/z	Ret. Time (min)	HMDB ID	Metabolites	POS/NEG	Formula	CON VS. MOD			NKT VS. MOD		
							p-value	VIP	FC	p-value	VIP	FC
60	202.1054	3.31	HMDB0001539	Asymmetric dimethylarginine	NEG	C8H18N4O2	7.1E-08	1.005	4.42629	0.00189	1.1361	2.15148
61	204.0646	4.81	HMDB0000929	L-Tryptophan	NEG	C11H12N2O2	3.4E-08	9.399	7.72363	1.6E-05	9.6808	1.82429
62	204.9799	3.64	HMDB0011621	Cinnamoylglycine	NEG	C11H11NO3	1.3E-05	1.2131	3.2584	0.00036	1.436	1.85785
63	207.0824	4.58	HMDB0000512	N-Acetyl-L-phenylalanine	NEG	C11H13NO3	0.00022	1.2431	1.38347	2.1E-06	1.8663	1.34117
64	210.0408	1.12	HMDB0000663	Glucaric acid	NEG	C6H10O8	4.9E-05	1.1774	6.39003	0.00064	1.5211	3.56113
65	210.9959	3.92	HMDB0001434	3-Methoxytyrosine	NEG	C10H13NO4	6.5E-07	1.1971	4.57633	0.00054	1.1907	1.68996
66	215.0336	5.93	HMDB0001885	3-Chlorotyrosine	NEG	C9H10ClNO3	3.5E-08	1.295	4.83498	3.2E-07	1.6565	2.58763
67	216.0835	2.9	HMDB0004620	N-a-Acetyl-L-arginine	NEG	C8H16N4O3	8.8E-08	1.0069	3.05661	0.00169	1.057	1.68557
68	216.0954	1.13	HMDB0000888	Undecanedioic acid	NEG	C11H20O4	0.00022	1.0216	8.67996	0.00602	1.0986	2.27087
69	220.0575	4.77	HMDB0000472	5-Hydroxy-L-tryptophan	NEG	C11H12N2O3	9.5E-07	1.803	8.00004	0.00016	1.9066	1.96717
70	221.0742	5.29	HMDB0000215	N-Acetyl-D-glucosamine	NEG	C8H15NO6	5.4E-08	1.0105	2.70659	0.00041	1.0063	1.48427
71	223.9976	3.89	HMDB0000732	Hydroxykynurenine	NEG	C10H12N2O4	3.3E-07	3.1913	8.39051	0.00013	3.2359	1.79902
72	227.1246	6.17	HMDB0000014	Deoxycytidine	NEG	C9H13N3O4	1.3E-05	2.0732	1.94613	0.00248	2.2009	1.42872
73	228.0836	3.5	HMDB0006695	Prolylhydroxyproline	NEG	C10H16N2O4	0.00122	1.2061	1.63125	0.00068	1.8084	1.59993
74	240.0289	4.76	HMDB0000192	L-Cystine	NEG	C6H12N2O4S2	1.9E-09	1.2397	5.99187	1.2E-05	1.2461	1.67187
75	242.0471	3.65	HMDB0000273	Thymidine	NEG	C10H14N2O5	8.1E-05	1.3176	4.83607	0.00984	1.2557	1.73544
76	243.059	1.01	HMDB0000089	Cytidine	NEG	C9H13N3O5	2.5E-08	3.4302	3.97544	0.00335	2.5211	1.30638
77	244.1157	4.64	HMDB0000767	Pseudouridine	NEG	C9H12N2O6	1.9E-09	1.288	3.03213	0.00015	1.4306	1.70518
78	246.012	3.88	HMDB0013713	N-Acetyltryptophan	NEG	C13H14N2O3	1.1E-07	1.3575	5.50081	0.00477	1.0292	1.40158
79	251.0919	5.49	HMDB0000101	Deoxyadenosine	NEG	C10H13N5O3	1.1E-05	1.8817	17.5767	0.00148	1.9545	2.19859

NO.	m/z	Ret. Time (min)	HMDB ID	Metabolites	POS/NEG	Formula	CON VS. MOD			NKT VS. MOD		
							p-value	VIP	FC	p-value	VIP	FC
80	255.1572	7.28	HMDB0002275	7,8-Dihydroneopterin	NEG	C9H13N5O4	1.7E-06	1.8926	2.33756	8.7E-05	2.3435	1.74522
81	257.0995	3.15	HMDB0000086	Glycerophosphocholine	NEG	C8H20NO6P	1.9E-06	1.6873	2.61309	0.00117	1.9761	1.70888
82	257.1721	7.69	HMDB0000982	5-Methylcytidine	NEG	C10H15N3O5	2.7E-05	1.4505	1.99452	0.00033	1.9017	1.70269
83	260.0211	1.13	HMDB0001401	Glucose 6-phosphate	NEG	C6H13O9P	1.1E-08	1.4982	5.28314	0.00062	1.1777	1.35987
84	262.0044	3.25	HMDB0011719	Homovanillic acid sulfate	NEG	C9H10O7S	1.4E-09	1.3954	11.7552	1.3E-05	1.4311	1.86265
85	264.0101	1.96	HMDB0006344	Phenylacetylglutamine	NEG	C13H16N2O4	1.1E-06	1.3461	2.959	0.00035	1.5661	1.77141
86	267.1198	7.03	HMDB0000050	Adenosine	NEG	C10H13N5O4	1.3E-11	1.4371	5.23901	1.5E-06	1.6443	2.00233
87	275.0926	5.47	HMDB0011738	N2-gamma-Glutamylglutamine	NEG	C10H17N3O6	3.4E-12	2.6725	4.73575	4.7E-06	2.9092	1.78026
88	276.087	4.64	HMDB0001316	6-Phosphogluconic acid	NEG	C6H13O10P	4.4E-10	1.0558	8.07816	5.3E-06	1.2078	2.19917
89	283.0793	4.34	HMDB0000133	Guanosine	NEG	C10H13N5O5	5.9E-08	8.6606	5.23434	0.00014	8.2122	1.5498
90	284.0215	3.19	HMDB0000299	Xanthosine	NEG	C10H12N4O6	7.4E-06	1.4206	25.0618	0.00117	1.5211	2.26149
91	285.1293	4.16	HMDB0005923	N4-Acetylcytidine	NEG	C11H15N3O6	6.6E-06	1.4786	5.16317	0.01276	1.313	1.56155
92	309.0938	4.96	HMDB0000230	N-Acetylneuraminic acid	NEG	C11H19NO9	1.1E-08	1.1354	18.0959	7.9E-05	1.1631	1.98193
93	311.0373	5.94	HMDB0004824	N2,N2-Dimethylguanosine	NEG	C12H17N5O5	6.5E-12	1.5465	16.3309	0.00981	1.0481	1.40044
94	329.0998	5.12	HMDB0000058	Cyclic AMP	NEG	C10H12N5O6P	6.6E-09	1.1705	2.71946	7.2E-08	1.5364	2.05921
95	345.0314	3.27	HMDB0001314	Cyclic GMP	NEG	C10H12N5O7P	3.9E-09	1.4513	9.28436	2.2E-07	1.8065	2.80144

Table S4 Differential metabolites in plasma after AOP-NKT treatment.

NO.	m/z	Ret. Time (min)	HMDB ID	Metabolites	POS/NEG	Formula	CON VS. MOD			AOP-NKT VS. MOD		
							p-value	VIP	FC	p-value	VIP	FC
1	88.047	5.67	HMDB0000243	Pyruvic acid	POS	C3H4O3	0.0001	1.4541	2.20999	0.00024	1.8948	1.89885
2	88.1569	5.65	HMDB0001414	Putrescine	POS	C4H12N2	5.3E-05	1.9773	2.37596	0.00021	2.5677	2.01726
3	89.0221	5.6	HMDB0000056	beta-Alanine	POS	C3H7NO2	8.7E-06	1.0706	3.71076	0.00089	1.2086	2.14309
4	118.0649	3.38	HMDB0000043	Betaine	POS	C5H12NO2	7E-06	1.1738	0.62415	2.2E-05	1.6948	0.61681
5	130.0866	1.03	HMDB0002064	N-Acetylputrescine	POS	C6H14N2O	1.1E-09	2.2331	7.18581	0.0059	1.4396	1.30116
6	142.1591	5.35	HMDB0240650	Ectoine	POS	C6H10N2O2	3E-05	1.3222	0.69253	5.2E-05	2.0912	0.64835
7	194.0818	4.24	HMDB0002545	Galacturonic acid	POS	C6H10O7	1.2E-05	2.1224	11.8496	0.00184	2.1751	2.16185
8	205.0986	3.3	HMDB0000671	Indolelactic acid	POS	C11H11NO3	0.01645	1.8184	1.81588	0.03086	2.3946	1.7223
9	240.094	1.12	HMDB0061112	3-Carboxy-4-methyl-5-propyl-2-furanpropionic acid	POS	C12H16O5	3.1E-05	1.0492	8.44132	0.001	1.1563	2.495
10	245.2268	14.93	HMDB0000688	Isovalerylcarnitine	POS	C12H23NO4	5.8E-07	2.3847	3.21978	7.2E-06	3.0273	2.36195
11	247.1067	4.75	HMDB0002095	Malonylcarnitine	POS	C10H17NO6	3.9E-07	1.3821	7.99286	0.00124	1.3161	1.83374
12	276.1272	3.86	HMDB0000279	Saccharopine	POS	C11H20N2O6	0.00023	1.067	4.76815	0.00239	1.1967	2.4111
13	285.2411	13.4	HMDB0013324	2-Octenoylcarnitine	POS	C15H27NO4	4.8E-05	1.3373	3.8453	0.00363	1.4537	1.95568
14	286.3085	8.75	HMDB0000305	Vitamin A(retinol)	POS	C20H30O	0.00017	1.0052	4.14085	0.00067	1.3216	2.84583
15	290.2651	6.79	HMDB0002961	Dihydrotestosterone	POS	C19H30O2	0.00062	1.8055	2.84883	0.0455	1.5342	1.55898
16	300.201	3.48	HMDB0001852	all-trans-Retinoic acid	POS	C20H28O2	6.7E-05	2.6476	2.85961	0.0002	3.3865	2.28499
17	368.2795	7.09	HMDB0002003	Tetracosanoic acid	POS	C24H48O2	0.00066	1.3988	1.77583	3.3E-05	2.3008	2.20446
18	374.3621	8.71	HMDB0000308	3b-Hydroxy-5-cholenoic acid	POS	C24H38O3	0.0002	3.3984	3.40203	0.00443	3.9738	2.07175

NO.	m/z	Ret. Time (min)	HMDB ID	Metabolites	POS/NEG	Formula	CON VS. MOD			AOP-NKT VS. MOD		
							p-value	VIP	FC	p-value	VIP	FC
19	379.2824	14.82	HMDB0000277	Sphingosine 1-phosphate	POS	C18H38NO5P	9E-08	2.1523	6.16406	1.7E-05	2.4678	2.35539
20	386.2912	6.78	HMDB0000067	Cholesterol	POS	C27H46O	0.00057	1.4173	3.69099	0.0034	1.7217	2.39436
21	390.3568	7.6	HMDB0000328	12-Ketodeoxycholic acid	POS	C24H38O4	7.7E-05	2.2667	2.96803	0.00465	2.5606	1.89875
22	396.3098	7.82	HMDB0002356	Hexacosanoic acid	POS	C26H52O2	0.00701	1.3929	2.01529	0.00092	2.3621	2.83682
23	402.3927	10.51	HMDB0001496	7alpha-Hydroxycholesterol	POS	C27H46O2	0.00042	2.0184	3.47125	0.00151	2.6818	2.67064
24	406.3525	6.81	HMDB0000391	7-Ketodeoxycholic acid	POS	C24H38O5	1.5E-06	3.3149	2.50582	0.01127	2.851	1.42843
25	730.539	14.71	HMDB0001348	SM(d18:1/18:0)	POS	C41H83N2O6P	0.00112	1.3496	1.78329	0.02132	1.4062	1.42116
26	103.0384	1.67	HMDB0000092	Dimethylglycine	NEG	C4H9NO2	2.2E-07	4.0548	1.88714	0.0012	3.7862	1.75176
27	109.0274	3.43	HMDB0000965	Hypotaurine	NEG	C2H7NO2S	0.01212	1.0799	9.57374	0.02628	1.0078	4.2942
28	111.0079	1.13	HMDB0000630	Cytosine	NEG	C4H5N3O	7.1E-10	4.1004	3.00577	1.3E-05	3.9619	2.40849
29	111.9443	3.96	HMDB0000300	Uracil	NEG	C4H4N2O2	7.6E-09	2.4341	4.63251	2.9E-08	2.483	3.5974
30	117.0537	3.06	HMDB0000532	Acetylglycine	NEG	C4H7NO3	1.8E-09	6.1226	2.34262	1.2E-07	7.4091	3.87261
31	117.0541	2.78	HMDB0000738	Indole	NEG	C8H7N	2.9E-07	2.2145	2.95742	2.7E-08	2.7737	10.051
32	119.0327	1.01	HMDB0000167	L-Threonine	NEG	C4H9NO3	6.2E-07	2.0151	2.46711	0.00016	2.0719	2.47899
33	124.9887	0.91	HMDB0000251	Taurine	NEG	C2H7NO3S	4.5E-08	1.9706	8.1101	0.00018	1.4906	1.96749
34	129.0172	1.12	HMDB0000267	Pyroglutamic acid	NEG	C5H7NO3	2.5E-09	2.6222	3.31722	9.1E-07	2.6536	2.82341
35	131.0706	4.21	HMDB0013222	Guanidinopropionic acid	NEG	C4H9N3O2	2.8E-08	5.2289	2.79646	1.3E-07	5.9227	3.58311
36	135.0268	0.93	HMDB0000034	Adenine	NEG	C5H5N5	1.6E-08	2.8567	2.34165	0.00368	2.226	1.63972
37	142.0491	1.75	HMDB0000469	5-Hydroxymethyluracil	NEG	C5H6N2O3	3.7E-09	2.2902	4.16348	4.9E-08	2.5437	5.96648
38	144.0424	3.4	HMDB0000522	3-Methylglutaconic acid	NEG	C6H8O4	5.7E-08	1.1907	3.35026	6.3E-05	1.15	2.62297

NO.	m/z	Ret. Time (min)	HMDB ID	Metabolites	POS/NEG	Formula	CON VS. MOD			AOP-NKT VS. MOD		
							p-value	VIP	FC	p-value	VIP	FC
39	144.064	1.94	HMDB0000482	Caprylic acid	NEG	C8H16O2	1.6E-07	2.588	3.2402	6.3E-08	3.2109	14.0853
40	146.9601	4.02	HMDB0000148	Glutamic acid	NEG	C5H9NO4	9.1E-05	1.2433	0.47233	6E-05	1.4687	0.41948
41	149.0432	0.98	HMDB0000696	Methionine	NEG	C5H11NO2S	2.2E-09	2.4596	15.8442	5.8E-06	2.1187	2.76973
42	151.0418	3.06	HMDB0000132	Guanine	NEG	C5H5N5O	2.3E-08	1.0485	5.68105	4.8E-07	1.0472	3.74082
43	155.1053	5.28	HMDB0000177	Histidine	NEG	C6H9N3O2	4.6E-11	1.914	5.96557	8.4E-06	1.8827	4.04998
44	156.0651	3.35	HMDB0000226	Orotic acid	NEG	C5H4N2O4	7.8E-08	1.8211	3.46542	1.6E-07	2.0895	5.87876
45	157.0341	0.96	HMDB0000959	Tiglylglycine	NEG	C7H11NO3	4.3E-11	3.253	4.9794	3.4E-05	2.6615	2.03595
46	157.0474	3.4	HMDB0000459	3-Methylcrotonylglycine	NEG	C7H11NO3	2.6E-09	1.104	4.03587	8.7E-09	1.2004	4.73711
47	158.0601	4.78	HMDB0000462	Allantoin	NEG	C4H6N4O3	4.4E-07	1.2829	11.0202	5.3E-06	1.2679	5.07031
48	158.0807	3.47	HMDB0003349	L-Dihydroorotic acid	NEG	C5H6N2O4	1.4E-06	2.8395	2.63979	1.7E-05	3.1873	3.43901
49	158.9759	0.79	HMDB0001190	Indole-3-acetaldehyde	NEG	C10H9NO	2.8E-05	2.0813	2.18292	0.04241	1.1334	1.28483
50	161.0431	1.15	HMDB0000510	Aminoadipic acid	NEG	C6H11NO4	7E-06	1.3075	1.79494	1E-06	1.5484	2.19306
51	162.0037	9.21	HMDB0000355	3-Hydroxymethylglutaric acid	NEG	C6H10O5	0.00068	1.2432	11.4778	0.01181	1.0036	2.66451
52	164.0701	4.6	HMDB0000205	Phenylpyruvic acid	NEG	C9H8O3	1.3E-09	1.0645	5.56065	2.5E-07	1.0673	3.76766
53	165.0535	4.6	HMDB0000897	7-Methylguanine	NEG	C6H7N5O	3.5E-06	2.8093	7.66639	2.3E-05	2.7569	4.18381
54	166.0158	0.98	HMDB0000779	Phenyllactic acid	NEG	C9H10O3	3.6E-08	3.3073	3.60188	5.3E-06	3.3292	3.02558
55	172.9891	4.93	HMDB0000842	Quinaldic acid	NEG	C10H7NO2	4.4E-08	1.6901	3.84046	2E-07	1.7731	3.58708
56	173.0051	1.13	HMDB0000701	Hexanoylglycine	NEG	C8H15NO3	1.3E-11	4.7558	4.2072	2.2E-06	4.5195	2.72639
57	174.0081	1.12	HMDB0060015	Phenol sulphate	NEG	C6H6O4S	3.5E-08	1.0216	3.05258	3.3E-05	1.0349	2.86064
58	175.0028	3.59	HMDB0000812	N-Acetyl-L-aspartic acid	NEG	C6H9NO5	4E-08	1.4571	3.25958	1.9E-07	1.6298	4.32866

NO.	m/z	Ret. Time (min)	HMDB ID	Metabolites	POS/NEG	Formula	CON VS. MOD			AOP-NKT VS. MOD		
							p-value	VIP	FC	p-value	VIP	FC
59	175.021	1.11	HMDB0000197	Indoleacetic acid	NEG	C10H9NO2	6.2E-06	1.5257	2.19959	3.1E-05	1.6466	2.29901
60	175.0217	4.31	HMDB0000904	Citrulline	NEG	C6H13N3O3	1.2E-08	1.3519	5.58348	2.3E-05	1.3082	3.48791
61	178.0476	3.86	HMDB0003466	L-Gulonolactone	NEG	C6H10O6	0.00017	2.0534	2.44232	0.00115	1.7762	1.76683
62	179.0533	0.97	HMDB0000714	Hippuric acid	NEG	C9H9NO3	4E-08	2.4338	2.58935	7E-05	2.2805	2.05049
63	181.0481	3.28	HMDB0000158	L-Tyrosine	NEG	C9H11NO3	2.7E-07	5.2049	5.33656	1.1E-06	5.5433	5.59261
64	182.0513	3.28	HMDB0000107	Galactitol	NEG	C6H14O6	4.7E-07	1.6615	5.4489	1.5E-06	1.7758	5.92128
65	184.1018	4.55	HMDB0001565	Phosphorylcholine	NEG	C5H15NO4P	4E-09	1.3069	3.10352	7E-07	1.4438	4.01665
66	185.0788	4.85	HMDB0000272	Phosphoserine	NEG	C3H8NO6P	4E-10	1.3456	3.80656	1E-05	1.3328	3.09974
67	186.0731	1.52	HMDB0000807	3-Phosphoglyceric acid	NEG	C3H7O7P	0.00058	1.0495	8.50059	0.00074	1.1289	7.59023
68	188.0676	5.87	HMDB0011635	p-Cresol sulfate	NEG	C7H8O4S	0.0044	1.0933	3.50225	0.01276	1.0166	2.56603
69	188.983	3.43	HMDB0001138	N-Acetyl-L-glutamic acid	NEG	C7H11NO5	0.00845	5.5893	8.66273	0.01944	5.2243	4.12605
70	189.057	5.94	HMDB0000715	Kynurenic acid	NEG	C10H7NO3	5.8E-08	1.3299	8.25313	8.8E-06	1.2632	3.59777
71	190.0032	4.46	HMDB0001370	Diaminopimelic acid	NEG	C7H14N2O4	1E-06	1.1386	4.76352	3.3E-05	1.1415	3.56495
72	191.0167	5.56	HMDB0011745	N-Acetyl-L-methionine	NEG	C7H13NO3S	2.7E-10	2.3417	2.36633	1.5E-05	2.1793	1.89272
73	192.0645	4.25	HMDB0000193	Isocitric acid	NEG	C6H8O7	8.1E-08	7.3577	5.7239	1.1E-06	7.4186	3.95237
74	193.0657	4.25	HMDB0000821	Phenylacetylglycine	NEG	C10H11NO3	0.00019	2.7346	6.51907	0.00052	2.7263	4.29095
75	194.0428	3.21	HMDB0000127	D-Glucuronic acid	NEG	C6H10O7	9.3E-09	1.4575	11.5523	4.6E-06	1.3857	4.13168
76	195.0486	0.91	HMDB0013678	p-Hydroxyhippuric acid	NEG	C9H9NO4	1E-07	3.6897	5.84948	0.0082	2.1068	1.46909
77	197.0791	4.8	HMDB0032055	N-Acetylhistidine	NEG	C8H11N3O3	1.1E-05	2.2175	2.3763	8.1E-06	2.6426	3.61995
78	201.0211	5.27	HMDB0000731	Cysteine-S-sulfate	NEG	C3H7NO5S2	1.2E-07	8.4799	11.9567	0.01311	5.3843	1.85026

NO.	m/z	Ret. Time (min)	HMDB ID	Metabolites	POS/NEG	Formula	CON VS. MOD			AOP-NKT VS. MOD		
							p-value	VIP	FC	p-value	VIP	FC
79	202.1054	3.31	HMDB0001539	Asymmetric dimethylarginine	NEG	C8H18N4O2	7.1E-08	1.005	4.42629	7.8E-07	1.0833	4.9527
80	204.0646	4.81	HMDB0000929	L-Tryptophan	NEG	C11H12N2O2	3.4E-08	9.399	7.72363	1.7E-06	9.353	4.51725
81	204.9418	6.8	HMDB0000881	Xanthurenic acid	NEG	C10H7NO4	0.00056	1.5493	9.17538	0.00831	1.405	3.71934
82	204.9799	3.64	HMDB0011621	Cinnamoylglycine	NEG	C11H11NO3	1.3E-05	1.2131	3.2584	1E-05	1.3249	3.59803
83	210.0408	1.12	HMDB0000663	Glucaric acid	NEG	C6H10O8	4.9E-05	1.1774	6.39003	0.00011	1.2081	5.33691
84	210.9959	3.92	HMDB0001434	3-Methoxytyrosine	NEG	C10H13NO4	6.5E-07	1.1971	4.57633	3.2E-06	1.1909	3.2808
85	215.0336	5.93	HMDB0001885	3-Chlorotyrosine	NEG	C9H10ClNO3	3.5E-08	1.295	4.83498	4.2E-06	1.3159	4.03599
86	216.0835	2.9	HMDB0004620	N-a-Acetyl-L-arginine	NEG	C8H16N4O3	8.8E-08	1.0069	3.05661	8E-08	1.1918	5.93028
87	220.0575	4.77	HMDB0000472	5-Hydroxy-L-tryptophan	NEG	C11H12N2O3	9.5E-07	1.803	8.00004	5.8E-06	1.8295	5.28564
88	221.0742	5.29	HMDB0000215	N-Acetyl-D-glucosamine	NEG	C8H15NO6	5.4E-08	1.0105	2.70659	2.9E-05	1.0467	2.73611
89	223.9976	3.89	HMDB0000732	Hydroxykynurenine	NEG	C10H12N2O4	3.3E-07	3.1913	8.39051	6.4E-06	3.1685	4.48707
90	227.1246	6.17	HMDB0000014	Deoxycytidine	NEG	C9H13N3O4	1.3E-05	2.0732	1.94613	0.00019	2.4987	2.95
91	228.0836	3.5	HMDB0006695	Prolylhydroxyproline	NEG	C10H16N2O4	0.00122	1.2061	1.63125	0.00061	1.6591	2.75284
92	240.0289	4.76	HMDB0000192	L-Cystine	NEG	C6H12N2O4S2	1.9E-09	1.2397	5.99187	2.6E-06	1.2272	3.86418
93	242.0471	3.65	HMDB0000273	Thymidine	NEG	C10H14N2O5	8.1E-05	1.3176	4.83607	0.00011	1.3912	4.95807
94	243.059	1.01	HMDB0000089	Cytidine	NEG	C9H13N3O5	2.5E-08	3.4302	3.97544	0.00061	2.7927	2.01782
95	244.0232	1.96	HMDB0000296	Uridine	NEG	C9H12N2O6	4.9E-07	1.3769	10.3279	1.7E-06	1.4878	12.3291
96	244.1157	4.64	HMDB0000767	Pseudouridine	NEG	C9H12N2O6	1.9E-09	1.288	3.03213	3.9E-06	1.4236	3.92542
97	246.012	3.88	HMDB0013713	N-Acetyltryptophan	NEG	C13H14N2O3	1.1E-07	1.3575	5.50081	2.3E-05	1.1881	2.41293
98	251.0919	5.49	HMDB0000101	Deoxyadenosine	NEG	C10H13N5O3	1.1E-05	1.8817	17.5767	4.1E-05	1.9022	7.75881

NO.	m/z	Ret. Time (min)	HMDB ID	Metabolites	POS/NEG	Formula	CON VS. MOD			AOP-NKT VS. MOD		
							p-value	VIP	FC	p-value	VIP	FC
99	255.1572	7.28	HMDB0002275	7,8-Dihydroneopterin	NEG	C9H13N5O4	1.7E-06	1.8926	2.33756	7.5E-06	2.2869	4.00494
100	257.0995	3.15	HMDB0000086	Glycerophosphocholine	NEG	C8H20NO6P	1.9E-06	1.6873	2.61309	3.1E-05	1.9045	3.35245
101	257.1721	7.69	HMDB0000982	5-Methylcytidine	NEG	C10H15N3O5	2.7E-05	1.4505	1.99452	5.3E-06	1.9194	4.13603
102	260.0211	1.13	HMDB0001401	Glucose 6-phosphate	NEG	C6H13O9P	1.1E-08	1.4982	5.28314	6.9E-05	1.1877	1.95765
103	262.0044	3.25	HMDB0011719	Homovanillic acid sulfate	NEG	C9H10O7S	1.4E-09	1.3954	11.7552	7E-07	1.3322	3.9835
104	264.0101	1.96	HMDB0006344	Phenylacetylglutamine	NEG	C13H16N2O4	1.1E-06	1.3461	2.959	9.9E-06	1.4867	3.6251
105	267.1198	7.03	HMDB0000050	Adenosine	NEG	C10H13N5O4	1.3E-11	1.4371	5.23901	3.7E-07	1.4795	4.379
106	275.0926	5.47	HMDB0011738	N2-gamma-Glutamylglutamine	NEG	C10H17N3O6	3.4E-12	2.6725	4.73575	7.5E-09	2.8585	4.94933
107	276.087	4.64	HMDB0001316	6-Phosphogluconic acid	NEG	C6H13O10P	4.4E-10	1.0558	8.07816	5.2E-07	1.0673	5.13921
108	281.0518	1.06	HMDB0003331	1-Methyladenosine	NEG	C11H15N5O4	6.6E-06	1.193	3.09737	0.00044	1.1391	2.39775
109	283.0793	4.34	HMDB0000133	Guanosine	NEG	C10H13N5O5	5.9E-08	8.6606	5.23434	4.5E-05	8.3949	3.45079
110	284.0215	3.19	HMDB0000299	Xanthosine	NEG	C10H12N4O6	7.4E-06	1.4206	25.0618	6.2E-05	1.3995	5.84744
111	285.1293	4.16	HMDB0005923	N4-Acetylcytidine	NEG	C11H15N3O6	6.6E-06	1.4786	5.16317	2.7E-05	1.5598	4.69229
112	297.097	4.93	HMDB0001563	1-Methylguanosine	NEG	C11H15N5O5	9.4E-07	1.6237	2.85794	0.03338	1.0825	1.66592
113	309.0739	3.71	HMDB0010319	Indoxyl glucuronide	NEG	C14H15NO7	9.9E-09	1.1017	4.2134	3.6E-05	1.095	3.40466
114	309.0938	4.96	HMDB0000230	N-Acetylneuraminic acid	NEG	C11H19NO9	1.1E-08	1.1354	18.0959	6.1E-08	1.17	8.29323
115	311.0373	5.94	HMDB0004824	N2,N2-Dimethylguanosine	NEG	C12H17N5O5	6.5E-12	1.5465	16.3309	1.2E-06	1.4203	3.56086
116	329.0998	5.12	HMDB0000058	Cyclic AMP	NEG	C10H12N5O6P	6.6E-09	1.1705	2.71946	1.6E-07	1.3868	4.84983
117	345.0314	3.27	HMDB0001314	Cyclic GMP	NEG	C10H12N5O7P	3.9E-09	1.4513	9.28436	3.7E-07	1.4795	5.76938
118	427.0025	1.11	HMDB0001341	Adenosine diphosphate	NEG	C10H15N5O10P2	2.4E-06	2.0536	3.68879	0.00022	2.0391	3.15238
